# RLAnOxPeptide: an integrated framework combining transformer and reinforcement learning for efficient antioxidant peptide prediction and innovative design

**DOI:** 10.1093/bioinformatics/btag504

**Published:** 2026-07-08

**Authors:** Changsheng Han, Jianda Yue, Yaqi Li, Huanyu Li, Hua Tan, Zhenyu Wang, Zhihan Qi, Junbao Zhou, Zhonghua Liu, Ying Wang

**Affiliations:** The National and Local Joint Engineering Laboratory of Animal Peptide Drug Development, College of Life Sciences, Hunan Normal University, Changsha, Hunan 410081, China; Peptide and Small Molecule Drug R&D platform, Furong Laboratory, Hunan Normal University, Changsha, Hunan 410081, China; Institute of Interdisciplinary Studies, Hunan Normal University, Changsha, Hunan 410081, China; The National and Local Joint Engineering Laboratory of Animal Peptide Drug Development, College of Life Sciences, Hunan Normal University, Changsha, Hunan 410081, China; Peptide and Small Molecule Drug R&D platform, Furong Laboratory, Hunan Normal University, Changsha, Hunan 410081, China; Institute of Interdisciplinary Studies, Hunan Normal University, Changsha, Hunan 410081, China; The National and Local Joint Engineering Laboratory of Animal Peptide Drug Development, College of Life Sciences, Hunan Normal University, Changsha, Hunan 410081, China; Peptide and Small Molecule Drug R&D platform, Furong Laboratory, Hunan Normal University, Changsha, Hunan 410081, China; Institute of Interdisciplinary Studies, Hunan Normal University, Changsha, Hunan 410081, China; The National and Local Joint Engineering Laboratory of Animal Peptide Drug Development, College of Life Sciences, Hunan Normal University, Changsha, Hunan 410081, China; Peptide and Small Molecule Drug R&D platform, Furong Laboratory, Hunan Normal University, Changsha, Hunan 410081, China; Institute of Interdisciplinary Studies, Hunan Normal University, Changsha, Hunan 410081, China; The National and Local Joint Engineering Laboratory of Animal Peptide Drug Development, College of Life Sciences, Hunan Normal University, Changsha, Hunan 410081, China; Peptide and Small Molecule Drug R&D platform, Furong Laboratory, Hunan Normal University, Changsha, Hunan 410081, China; Institute of Interdisciplinary Studies, Hunan Normal University, Changsha, Hunan 410081, China; The National and Local Joint Engineering Laboratory of Animal Peptide Drug Development, College of Life Sciences, Hunan Normal University, Changsha, Hunan 410081, China; The National and Local Joint Engineering Laboratory of Animal Peptide Drug Development, College of Life Sciences, Hunan Normal University, Changsha, Hunan 410081, China; The National and Local Joint Engineering Laboratory of Animal Peptide Drug Development, College of Life Sciences, Hunan Normal University, Changsha, Hunan 410081, China; The National and Local Joint Engineering Laboratory of Animal Peptide Drug Development, College of Life Sciences, Hunan Normal University, Changsha, Hunan 410081, China; Peptide and Small Molecule Drug R&D platform, Furong Laboratory, Hunan Normal University, Changsha, Hunan 410081, China; Institute of Interdisciplinary Studies, Hunan Normal University, Changsha, Hunan 410081, China; The National and Local Joint Engineering Laboratory of Animal Peptide Drug Development, College of Life Sciences, Hunan Normal University, Changsha, Hunan 410081, China; Peptide and Small Molecule Drug R&D platform, Furong Laboratory, Hunan Normal University, Changsha, Hunan 410081, China; Institute of Interdisciplinary Studies, Hunan Normal University, Changsha, Hunan 410081, China

## Abstract

**Motivation:**

Bioactive peptides exhibit immense potential in pharmaceutical and food science domains, with antioxidant peptides (AOPs) garnering significant attention for their roles in scavenging free radicals. However, traditional discovery methods are inefficient and costly. This study introduces RLAnOxPeptide, an integrated computational framework that merges machine learning and reinforcement learning for the efficient prediction and *de novo* design of AOPs.

**Results:**

The framework initially establishes a high-precision predictor, RLP-T5Pred, based on the ProtT5 model via a 'protein-to-peptide’ knowledge transfer strategy. By employing label smoothing and logit penalty regularization, it achieves state-of-the-art accuracy (AUC-ROC: 0.9692) and robust calibration. The second component is the generator, RLP-T5Gen, which is trained in an iterative 'Yin-Yang’ loop combining supervised learning (to maintain sequence syntax) and reinforcement learning (to drive innovation). Guided by RLP-T5Pred serving as a fixed evaluator and a multi-objective reward function, the generator efficiently designs novel AOPs with high predicted activity. We experimentally validated the framework by synthesizing 17 designed peptides. Most candidates demonstrated potent radical scavenging abilities in chemical assays (DPPH and ABTS), leading to the selection of the top five candidates for cellular validation. In a t-BHP-induced HepG2 cell model, peptides Pep4, Pep5, Pep10, and Pep11 exhibited significant protective effects against oxidative damage. Consequently, the RLAnOxPeptide framework provides a powerful, experimentally verified paradigm for accelerating the discovery of novel antioxidant peptides.

**Availability:**

The datasets generated and/or analysed during the current study, along with model outputs and representative peptide sequences, have been deposited in a public repository. The RLAnOxPeptide framework source code is available at GitHub: https://github.com/changshh/RLAnOxPeptide. An archival snapshot of the code used to perform the experiments described in this manuscript has been deposited in Zenodo with the DOI: 10.5281/zenodo.20078425. An interactive online demonstration is also available via Hugging Face Spaces: https://huggingface.co/spaces/chshan/RLAnOxPeptide.

## 1 Introduction

Oxidation reactions, as fundamental chemical processes in biological systems and the material world, demonstrate remarkable duality. On one hand, they are central to energy-producing pathways like cellular respiration (Hellwig 2019); on the other, uncontrolled oxidation leads to food spoilage (Hellwig 2019) and induces various human diseases (Wang *et al*. 2021). Within the body, although oxidation participates in energy metabolism and immune defense, the excessive accumulation of its byproducts reactive oxygen species (ROS) triggers oxidative stress. The latter is confirmed to be closely associated with cardiovascular diseases, neurodegenerative disorders, cancer, and the aging process (Herb and Schramm 2021), mechanistically linked to ROS-induced damage to macromolecules like cell membranes, proteins, and DNA (Rice-Evans and Diplock 1993).

Antioxidant peptides (AOPs), as a class of highly promising bioactive molecules, are becoming a focal point in research combating oxidative damage. These short peptides, typically composed of 2–20 amino acids, often originate from the enzymatic hydrolysis or fermentation of natural proteins (Sarmadi and Ismail 2010, Liu *et al*. 2016). Their small molecular weight (< 3–6 kDa) facilitates absorption and utilization. AOPs exert antioxidant functions by scavenging free radicals, inhibiting lipid peroxidation, and other mechanisms, often existing in an inactive form within the parent protein and requiring hydrolysis for release (Zhang *et al*. 2024). Their activity is intimately linked to amino acid composition, sequence, length, spatial conformation, hydrophobicity, and charge distribution (Olsen *et al.* 2020). Specific amino acids (e.g. hydrophobic, aromatic, sulfur-containing, acidic/basic residues) and their positions in the sequence (especially at the N/C-terminus) significantly influence the activity (Elias *et al.* 2008, Du *et al.* 2022).

As natural antioxidants, AOPs are highly favored for their excellent safety profile, high efficacy, and wide availability (Qin *et al*. 2023). They hold promise to replace synthetic antioxidants in food preservation (Zhang *et al*. 2024) and serve as functional ingredients in health products to modulate human oxidative stress (Zhang *et al*. 2024). Many food-derived peptides exhibit multiple biological effects alongside antioxidant activity, such as anti-hypertensive and anti-inflammatory properties, greatly expanding their application prospects (Zhu *et al.* 2022, 2024). However, traditional experiment-based AOP screening methods are time-consuming, expensive, and rely heavily on expert experience, severely limiting the rapid discovery of novel AOPs (Meng *et al.* 2023).

To overcome this bottleneck, computational methods, particularly machine learning (ML) and deep learning (DL), have emerged as powerful tools for predicting and designing AOPs (Geng *et al.* 2023). Leveraging resources from databases like BIOPEP-UWM (Minkiewicz *et al.* 2019, Cui *et al.* 2022), Peptipedia (Quiroz *et al.* 2021) and AODB (Deng *et al.* 2023), ML/DL models such as Convolutional Neural Networks (CNN), Recurrent Neural Networks (RNN), and Transformers (Zhang *et al*. 2024) can learn complex structure-activity relationships from sequence data, automatically extracting features and surpassing traditional QSAR models (Du *et al.* 2022). Effective peptide sequence representation (evolving from amino acid composition to pretrained language model embeddings (Gronning *et al.* 2021, Du *et al.* 2023)) is crucial for model performance.

Furthermore, research focus has shifted from activity prediction towards *de novo* peptide design using generative models (e.g. Variational Autoencoders (VAEs) (El Kojok 2025, Oulmalme *et al.* 2025), Generative Adversarial Networks (GANs) (Oulmalme *et al.* 2025), and diffusion models (Zhong *et al.* 2024) (Hesamzadeh *et al.* 2024), aiming to create entirely new sequences with high predicted activity (Oulmalme *et al.* 2025). Molecular docking and quantum chemistry calculations (like DFT) (Zhang *et al*. 2025a) are also employed for mechanism elucidation and optimization (Hesamzadeh *et al.* 2024).

Despite significant progress, ML/DL-based AOP research still faces key challenges that limit its practical application (Li *et al.* 2025b). Firstly, while numerous predictive models exist, there is still considerable room for improvement in their prediction accuracy and generalization ability, which are fundamental to reliably identifying promising candidates. Secondly, most research has focused on discriminating known peptides rather than designing entirely new ones. The ability to generate novel peptide sequences with high antioxidant potential from scratch remains a significant hurdle. Finally, a critical gap often persists between *in silico* predictions and real-world biological activity, highlighting the need for a framework that not only designs peptides computationally but also validates their efficacy experimentally (Zhang *et al*. 2025a).

To address these critical gaps, this study introduces RLAnOxPeptide, an integrated computational framework designed for both the highly accurate prediction and the innovative design of AOPs. RLAnOxPeptide comprises two core, synergistic components: one is a deeply optimized AOP predictor engineered for maximal accuracy and predictive reliability, the other is a reinforcement learning-driven peptide sequence generator that leverages the pretrained ProtT5 architecture. The model is also further enhanced by a unique training regimen that combines supervised learning (SL) with reinforcement learning (RL) co-training. This study goes beyond computational prediction by validating the framework through a comprehensive “dry-to-wet” loop. We synthesized 17 generated peptides and tested them using chemical antioxidant assays. Furthermore, we selected the most promising candidates for cellular assays. This multi-level validation confirms that RLAnOxPeptide not only generates chemically active peptides but also identifies candidates capable of protecting biological systems from oxidative injury.

## 2 Methods

### 2.1 Dataset construction and preprocessing

#### 2.1.1 Raw data collection

AOP data were primarily sourced from public databases DFBP (Qin *et al*. 2022), BIOPEP-UWM (Minkiewicz *et al.* 2019), Peptipedia (Quiroz *et al.* 2021), AODB (Deng *et al.* 2023), PlantPepDB (Das *et al*. 2020), FermFooDb (Chaudhary *et al*. 2021) and literature. After deduplication and removal of sequences overlapping with known non-active peptides, sequences with lengths between 2 and 20 amino acids were selected, yielding 1697 initial positive AOPs. Negative data were retrieved from the UniProt database via the UniProt REST API for peptides of 2–20 amino acids, excluding entries containing antioxidant-related keywords (antioxidant, antioxidative, anti-oxidant, anti-oxidative, oxidative stress, free radical, free radicals, radical scavenger, oxidant scavenger, reactive oxygen species, ROS clearance, ROS scavenger, ROS detoxification, ROS inhibition, metal chelation, metal chelator, metal-binding, and chelating activity). Sequences with non-standard amino acids or duplicates from the positive set were removed. Finally, a number of sequences equivalent to the positive dataset size were randomly selected as the initial negative set.

#### 2.1.2 Dataset cleaning

To enhance data purity, the CleanLab tool (Northcutt *et al.* 2021, Zhang *et al*. 2025b) was employed to identify and remove potential label noise. By employing the feature extraction method detailed in Section 2.2, CleanLab identified likely mislabeled samples. After removing this noise, the final dataset, containing 1528 AOPs (positive) and 1509 non-AOPs (negative), was first partitioned to reserve an independent test set (10% of the total data). The remaining 90% of the data was subsequently subdivided into a training set (90%) and a validation set (10%). UMAP (Armstrong *et al.* 2021) was used for dimensionality reduction and visualization of the cleaned data.

#### 2.1.3 Domain-adaptive fine-tuning of ProtT5 model

To adapt a powerful pretrained language model to the specific characteristics of antioxidant peptides, we selected Prot-T5_XL_UniRef50, a model also commonly known as ProtT5 (Elnaggar *et al.* 2022).

To specialize this general-purpose model, we performed domain-adaptive fine-tuning on our constructed antioxidant proteins dataset using a Masked Language Modeling (MLM) task. In this task, the model learned to predict randomly masked amino acids by optimizing the minimization of the prediction loss (Long *et al.* 2024):


(1) $$L_{\mathrm{MLM}}=-\sum_{i=1}^{d}\;l\mathrm{og}P(y_{i}|w_{i})$$

Where *d* is the total number of masked tokens, $y_{i}$ is the true value of the *i*th [MASK] token, and $w_{i}$ is the encoded representation of the *i*th [MASK] token. The detailed hyperparameters for fine-tuning are listed in Table S6, available as supplementary data at *Bioinformatics* online in Supplementary Information.

### 2.2 Feature engineering

#### 2.2.1 Fine-tuned ProtT5 embedding features

Following the domain-adaptive fine-tuning described above, this specialized ProtT5 model was then used to encode each peptide sequence into a fixed-length feature vector.

The encoding process is as follows: for a given peptide sequence with length *L*, the fine-tuned ProtT5 model first generates a per-residue embedding, resulting in a matrix with dimensions *L × 1024*. To create a single global representation for the entire peptide, which is necessary for downstream machine learning models, we apply mean pooling along the length dimension of the matrix. This aggregation step collapses the matrix into a final 1024-dimensional vector. This vector serves as the comprehensive, fine-tuned ProtT5 embedding feature, capturing the essential characteristics of the peptide for our analysis.

#### 2.2.2 Sequence feature extraction

To complement the deep learning embeddings, a set of traditional bioinformatics features known to be relevant to antioxidant activity was incorporated. These included compositional features such as Amino Acid Composition (AAC) (Chen *et al.* 2013) and Dipeptide Composition (DPC) (Liu *et al*. 2015); key physicochemical properties like the Grand Average of Hydropathicity (GRAVY) (Li 2024), Isoelectric Point (pI) (Reifenberg and Zimmer 2024), and Molecular Weight (MolWeight) (Liu *et al*. 2024); and redox-related features representing the peptide’s redox potential, such as the proportion of reducing amino acids (Zhou *et al*. 2024).

#### 2.2.3 Feature integration and normalization

The extracted features were first concatenated into a comprehensive raw feature vector. To mitigate the potential influence of outliers, the RobustScaler method from the Scikit-learn library was then applied to scale the data (Wongoutong 2024). This method centers features using the median and scales them by the interquartile range (IQR) (Jeng and Chen 2017), making it robust to outliers. This transformation yields the final input features, according to the formula:


(2) $$X_{i}^{\mathrm{scaled}}=\frac{X_{i}-\mathrm{median}(X)}{Q_{3}(X)-Q_{1}(X)}$$

Where $X_{i}^{\mathrm{scaled}}$ is the normalized value of the *i*th sample, $X_{i}$ is its original value, median(X) is the median of the feature vector, and $Q_{1}(X)$ and $Q_{3}(X)$ are the first (25th percentile) and third (75th percentile) quartiles of the feature vector, respectively. The denominator, $Q_{3}(X)-Q_{1}(X)$, represents the Interquartile Range (IQR) of the data.

### 2.3 Construction and optimization of the antioxidant peptide predictor (RLP-T5Pred)

The RLP-T5Pred predictor employs a sophisticated feature fusion architecture engineered for high-precision binary classification. The framework leverages a domain-adapted ProtT5 language model to extract deep contextual embeddings, which are concatenated with a vector of handcrafted physicochemical features. This fused vector v serves as the input to a Multilayer Perceptron (MLP) to compute the unnormalized logit z.

To ensure robustness and prevent the “overconfidence” often seen in neural networks—which is detrimental when used as a reward signal—we implemented a rigorous training objective incorporating Label Smoothing and Logit Penalty Regularization. Instead of minimizing the standard binary cross-entropy against hard targets y ∈{0, 1}, we optimize the following compound loss function $L_{\sup}$:


(3) $$L_{\sup}=\mathrm{BCE}(\hat{y},y_{ls})+\lambda_{\mathrm{reg}}\cdot \frac{1}{N}\sum_{i=1}^{N}\;z_{i}^{2}$$

Where $\hat{y}=\sigma (z)$ is the predicted probability, and $y_{ls}=y(1-\epsilon )+0.5\epsilon$ represents the smoothed labels with a smoothing factor ϵ=0.1. The second term is the L2 regularization of the logits (Logit Penalty) with weight $\lambda_{\mathrm{reg}}=0.05$, which prevents the logits z from growing indefinitely, thereby maintaining a calibratable probability distribution. Detailed training hyperparameters for RLP-T5Pred are provided in Table S7, available as supplementary data at *Bioinformatics* online in Supplementary Information.

Following convergence, we applied Temperature Scaling to calibrate the model’s output. We froze the model weights and learned a single scalar parameter T (temperature) by minimizing the negative log-likelihood on the validation set. The final calibrated probability $P_{\mathrm{cal}}$ is given by:


(4) $$P_{\mathrm{cal}}(y=1|v)=\sigma (\frac{z(v)}{T})=\frac{1}{1+\exp\left(-z(v)/T\right)}$$

This post-hoc calibration ensures that the output probabilities accurately reflect the true likelihood of antioxidant activity, providing a high-fidelity signal for the reinforcement learning phase.

### 2.4 Peptide sequence generator (RLP-T5Gen)

The RLP-T5Gen module is a Transformer-based autoregressive model designed for the *de novo* generation of novel peptides. The training paradigm embodies a “Yin-Yang” iterative loop that alternates between Supervised Learning (SL) and Reinforcement Learning (RL).

“Yin” Phase: Supervised Foundation. In the SL phase, the model minimizes the negative log-likelihood of the next amino acid prediction given the context, utilizing label smoothing to prevent overfitting to specific training sequences:


(5) $$L_{SL}=-\sum_{t}\log P(x_{t}|x_{<t};\theta )$$

This ensures the generator adheres to the fundamental grammatical rules of peptide sequences.

“Yang” Phase: Guided Innovation via Regularized REINFORCE.

In the RL phase, we treat sequence generation as a Markov Decision Process (MDP). To encourage the generation of novel, high-activity peptides while preventing mode collapse, we employ the REINFORCE policy gradient algorithm augmented with Entropy Regularization. The generator’s policy $\pi_{\theta }$ is updated to maximize the expected reward J(θ). The gradient estimate is computed as:


(6) $$\nabla_{\theta }J(\theta )\approx \frac{1}{B}\sum_{i=1}^{B}\sum_{t=1}^{L_{i}}\nabla_{\theta }\log\pi_{\theta }(x_{t}^{\left(i\right)}|x_{<t}^{\left(i\right)})\cdot (R(S^{\left(i\right)})-b)+\beta \nabla_{\theta }H(\pi_{\theta })$$

Where B is the batch size, $R(S^{\left(i\right)})$ is the cumulative reward for sequence $S^{\left(i\right)}$, and b is a moving average baseline used to reduce variance. Crucially, the entropy term $H(\pi_{\theta })$ with coefficient β (set to 0.025) encourages the model to maintain exploration and generate diverse sequences rather than converging to a single optimal solution. The specific hyperparameters used for generator training are listed in Table S8, available as supplementary data at *Bioinformatics* online in Supplementary Information.

The multi-objective reward function R(S) is defined as a weighted linear combination of functional and structural properties:


(7) $$R(S)=w_{\mathrm{clf}}\cdot \tilde{P_{\mathrm{cal}}}+w_{\mathrm{len}}\cdot e^{-\frac{{\left(L-\mu \right)}^{2}}{2\sigma^{2}}}+w_{\mathrm{div}}\cdot D_{\mathrm{seq}}+w_{\mathrm{nov}}\cdot I_{\mathrm{novel}}$$

Here, $\tilde{P_{\mathrm{cal}}}$ is the transformed predictor score, the second term is a Gaussian reward encouraging peptides near a target length μ, $D_{\mathrm{seq}}$ represents diversity metrics (unigram and bigram), and $I_{\mathrm{novel}}$ is an indicator function for novelty relative to the training set. This formulation precisely guides the generator towards the high-activity region of the chemical space while ensuring structural diversity.

### 2.5 Model evaluation metrics

#### 2.5.1 Evaluation of the predictor RLP-T5Pred

The standard binary classification metrics include Accuracy, Precision, Recall (Sensitivity), F1-Score, and AUC-ROC.


(8) $$\mathrm{Accuracy}=\frac{TP+TN}{TP+TN+FP+FN}$$

(9) $$\mathrm{Precision}=\frac{TP}{TP+FP}$$

(10) $$\mathrm{Recall}=\frac{TP}{TP+FN}$$

(11) $$F1=2\times \frac{\mathrm{Precision}\times \mathrm{Recall}}{\mathrm{Precision}+\mathrm{Recall}}$$

(12) $$\mathrm{AUC}=\int_{0}^{1}\;R\mathrm{OC}(t)dt$$

True Positives (TP) are actual AOPs correctly identified as AOPs.

True Negatives (TN) are non-AOPs correctly identified as non-AOPs.

False Positives (FP) are non-AOPs incorrectly identified as AOPs.

False Negatives (FN) are AOPs incorrectly identified as non-AOPs.

#### 2.5.2 Evaluation of the generator RLP-T5Gen

The quality of sequences generated by RLP-T5Gen was assessed using the following metrics, which align with the axes in the performance radar chart (Fig. 4C):

1. *Mean Predicted Activity*: The average predicted antioxidant activity score provided by the RLP-T5Pred classifier for all valid generated sequences.
2. *Median Predicted Activity*: The median value of the predicted activity scores, representing the central tendency of the generated library’s performance.
3. *Top-10% Activity*: The mean predicted activity score of the top 10% highest-scoring sequences, reflecting the model’s ability to generate elite candidates.
4. *Uniqueness* ($R_{\text{unique}}$*)*: The fraction of generated sequences that are unique (non-duplicate).
  (13) $$R_{\mathrm{unique}}=\frac{\text{Count}\;\text{of}\;\text{unique}\;\text{sequences}}{\text{Total}\;\text{number}\;\text{of}\;\text{generated}\;\text{sequences}}$$
5. *Shannon Entropy *(H): A measure of the global diversity of amino acid usage across all generated sequences.
  (14) $$H=-\sum_{x\in A}P(x)\log_{2}P(x)$$
  Where A is the set of 20 standard amino acids, and P(x) is the frequency of occurrence of amino acid x in the entire generated dataset.
6. *Unigram Diversity *($\bar{D}$): The average number of unique amino acid types per sequence, normalized by sequence length.
  (15) $$\bar{D}=\frac{1}{N}\sum_{i=1}^{N}\;D_{i}$$
   
  (16) $$D_{i}=\frac{\text{Count}\;\text{of}\;\text{unique}\;\text{amino}\;\text{acids}\;\text{in}\;S_{i}}{L_{i}}$$
7. *Bigram Diversity *($\bar{B}$): The average ratio of unique adjacent amino acid pairs (bigrams) to the total number of bigrams in a sequence.
  (17) $$\bar{B}=\frac{1}{N}\sum_{i=1}^{N}\;B_{i}$$
   
  (18) $${\;B}_{i}=\frac{\text{Count}\;\text{of}\;\text{unique}\;\text{bigrams}\;\text{in}\;S_{i}}{\text{Total}\;\text{number}\;\text{of}\;\text{bigrams}\;\text{in}\;S_{i}}$$

Note: In the formulas above, N is the total number of generated sequences, $S_{i}$ is the i-th sequence, and $L_{i}$ is the length of sequence$\;S_{i}$

### 2.6 Experimental validation

#### 2.6.1 Peptide synthesis and quality validation

All 17 candidate peptides were commercially synthesized by Nanjing Peptide Biotechnology Co., Ltd. (Nanjing, China) using standard 9-fluorenylmethoxycarbonyl (Fmoc) solid-phase peptide synthesis. The identity of each synthesized peptide was validated by mass spectrometry (MS), and peptide purity was confirmed by high-performance liquid chromatography (HPLC) by the synthesis company. Only peptides with confirmed HPLC purity ≥95% were used for subsequent antioxidant assays. The original mass spectrometry reports for all synthesized peptides are provided in Supplementary Figures S8–S24, available as supplementary data at *Bioinformatics* online. Lyophilized peptide powders were dissolved in phosphate-buffered saline (PBS) at the required concentrations before experimental use. All peptides were fully soluble in PBS at concentrations up to 2.0 mg/mL under the experimental conditions used in this study, and no visible precipitation or aggregation was observed.

#### 2.6.2 DPPH radical scavenging activity assay

The DPPH radical scavenging activity was measured using a commercial kit (T-AOC assay kit, DPPH method, Cat# D799296-0100, Sangon Biotech) with significant procedural optimizations determined through our preliminary explorations. To enable the peptides to achieve their maximum antioxidant potential, the reaction time was extended.

The optimized procedure was as follows: First, in individual Eppendorf (EP) tubes, 20 µL of peptide solution (or Trolox standard) was thoroughly mixed with 380 µL of the DPPH working solution. The EP tubes were then sealed and incubated for 24 h under dark, refrigerated (4°C) conditions to ensure the reaction reached completion. Critically, throughout this 24 h period, we ensured that the absorbance of the DPPH negative control (reagent without peptide) remained consistently unchanged, which confirms the stability of the reagent under these conditions and validates the accuracy of the endpoint measurements.

After the incubation was complete, 200 µL of the reaction mixture was transferred from each EP tube into a 96-well plate. The absorbance was immediately measured at 515 nm using a microplate reader. The ΔA was calculated as${\;A}_{\text{blank}}-A_{\text{test}}$. The total antioxidant capacity was calculated based on a Trolox standard curve (y=0.7072x-0.0081, $R^{2}=0.9977$) and expressed as mmol/L Trolox equivalents (TE). The calculation formula used was:

Total Antioxidant Capacity


(19) $$(µ\text{mol}\;\text{Trolox}/\text{mL})=\frac{\Delta A+0.0081}{0.7072}$$

Where x represents the Trolox concentration and y represents ΔA.

#### 2.6.3 ABTS radical cation scavenging activity assay

The ABTS assay was conducted using a commercial kit (T-AOC assay kit, ABTS method, Cat# D799298-0100, Sangon Biotech). The ABTS•+ radical cation working solution was prepared by mixing the kit’s stock solutions (7 mL of Reagent 1 added to Reagent 2 powder) and allowing them to react in the dark for 20 min to generate the stable blue-green radical cation, as per the manufacturer’s instructions.

The experimental procedure was as follows: First, in individual EP tubes, 10 µL of each peptide solution was mixed and thoroughly vortexed with 190 µL of the freshly prepared ABTS•+ working solution. The EP tubes were incubated at room temperature in the dark for 20 min. Following the incubation, an appropriate volume of the reaction mixture was transferred from each EP tube into a 96-well plate. The decrease in absorbance was immediately measured at 734 nm. The ΔA was calculated as $A_{\text{blank}}-A_{\text{test}}$. The antioxidant capacity was calculated from a Trolox standard curve ($y=0.55x+0.0006,R^{2}=0.999$) and expressed as mmol/L Trolox equivalents (TE). The calculation formula used was:


(20) $$\text{Total}\;\text{Antioxidant}\;\text{Capacity}\;(µ\text{mol}\;\text{Trolox}/\text{mL})=\frac{\Delta A-0.0006}{0.55}$$

Where x represents the Trolox concentration and y represents ΔA.

#### 2.6.4 Cell culture and reagents

The HepG2 cell line was selected for assessing the cellular antioxidant activity. Cells were cultured in high-glucose Dulbecco’s Modified Eagle Medium (DMEM; Cat. No. C11995500BT, Gibco) supplemented with 10% fetal bovine serum (FBS; Cat. No. 10099141C, Gibco) and 1% penicillin-streptomycin solution (Cat. No. P1400, Solarbio). The cultures were maintained at 37°C in a humidified incubator with 5% CO_2_. Phosphate-buffered saline (PBS; Cat. No. C10010500BT, Gibco) was used for cell washing.

#### 2.6.5 Cytoprotective activity assay (CCK-8 method)

To evaluate the ability of the designed peptides to protect cells against oxidative stress, a tert-butyl hydroperoxide (t-BHP; 70% solution in water, Cat. No. B111364, Aladdin) induced damage model was established. First, to determine the optimal damage condition, cells were treated with a concentration gradient of t-BHP (0–1000 µM) for varying durations (2, 4, and 6 h). Based on the results, treatment with 500 µM t-BHP for 4 h was selected for subsequent cytoprotection assays.

For the cytoprotection assay, cells were seeded in 96-well plates at a density of $5\times 10^{3}$ cells/well and allowed to adhere for 24 h. The cells were then pre-treated with the selected peptides (Pep4, Pep5, Pep10, and Pep11) at final concentrations of 0.25, 0.5, 1.0, and 2.0 mg/mL for 24 h. Following pretreatment, the medium was removed, and cells were exposed to t-BHP at the pre-determined IC_50_ concentration to induce oxidative damage. Cell viability was quantified using the Cell Counting Kit-8 (CCK-8; Cat. No. BA00208, Boaoshen). Absorbance was measured at 450 nm using a microplate reader.

The cell viability was calculated using the following formula:


(21) $$\text{Cell}\;\text{Viability}\;(\%)=\frac{A_{s}-A_{b}}{A_{c}-A_{b}}\times 100\%$$

Where $A_{s}$ is the absorbance of the experimental well (containing cells, CCK-8, and treatment), $A_{c}$ is the absorbance of the control well (containing cells and CCK-8, without treatment), and $A_{b}$ is the absorbance of the blank well (containing medium and CCK-8, without cells).

## 3 Results

**Figure 1:**
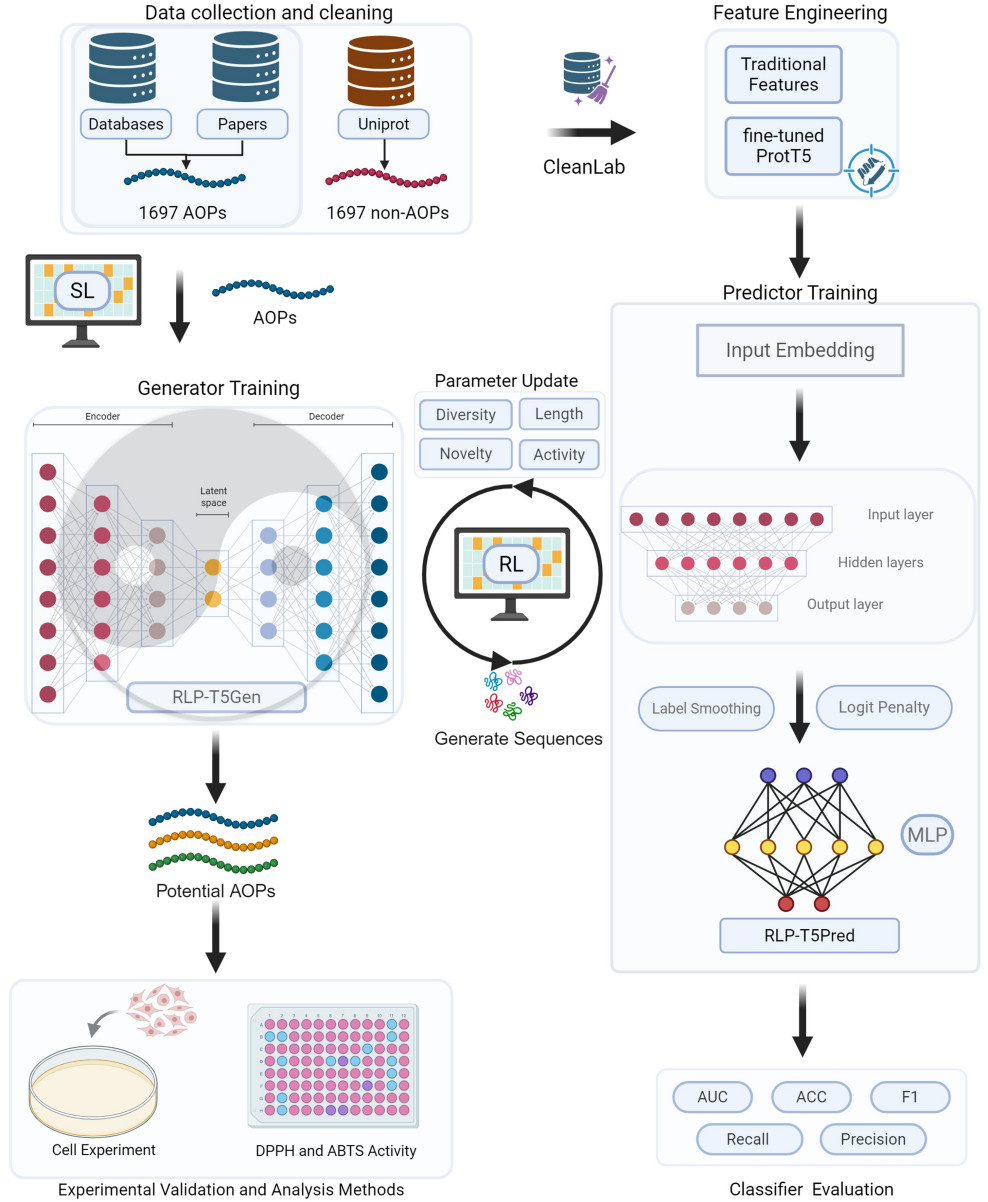
The overall architecture and workflow of the RLAnOxPeptide framework. (1) Data collection and cleaning: Antioxidant (AOP) and non-AOP sequences are collected from public databases and papers. The raw dataset is then purified using the CleanLab algorithm to identify and remove samples with potential label noise, yielding a high-quality dataset. (2) Feature Engineering: Two sets of features are generated from the clean data for the predictor model: a vector of traditional bioinformatics features (e.g. AAC, PPC) and deep representations derived from a domain-adapted, fine-tuned ProtT5 model. (3) Predictor Training (RLP-T5Pred): A Transformer-based predictor is trained on the engineered features. By leveraging supervised learning (SL) with label smoothing and logit penalty regularization. (4) Generator Training (RLP-T5Gen): A generative model, also built on the fine-tuned ProtT5 backbone, creates novel peptide sequences. Its training is guided by the previously trained predictor in an iterative “Yin-Yang” loop. This process integrates supervised learning on collected antioxidant peptides to ensure sequence validity with the REINFORCE algorithm to optimize a multi-objective reward function—balancing predicted activity, length, diversity, and novelty—which informs the generator’s policy updates. (5) Experimental Validation: High-potential peptides designed by the generator are selected for chemical synthesis. Their antioxidant capacities are first experimentally confirmed using DPPH and ABTS radical scavenging assays. Subsequently, candidates exhibiting superior activity are subjected to cellular experiments to verify their cytoprotective efficacy against oxidative damage.

The workflow of the RLAnOxPeptide framework is depicted in Fig. 1. The process began with data curation, where antioxidant (AOP) and non-AOP sequences were collected and purified with CleanLab to establish a high-quality training dataset. The foundational step was the domain-adaptive fine-tuning of the ProtT5 language model on a dataset of antioxidant proteins to create a specialized backbone. Subsequently, this specialized model serves as a base for two modules. The predictor, RLP-T5Pred, was trained on the curated peptide data using supervised learning to optimize its decision boundary and serve as a reliable evaluator. The generator, RLP-T5Gen, was also built upon the fine-tuned ProtT5 and acts as the creative engine. Guided by the trained classifier, it used the REINFORCE (Chan *et al*. 2024) algorithm to optimize a multi-objective reward function for activity, novelty, diversity, and length, thus producing potential AOPs. Finally, in the experimental validation phase, top-ranked candidate peptides were randomly selected and synthesized. Their radical scavenging capacities were assessed using DPPH and ABTS assays, and those demonstrating potent activity were selected for cellular experiments to confirm their intracellular efficacy.

### 3.1 Dataset construction and validation

**Figure 2:**
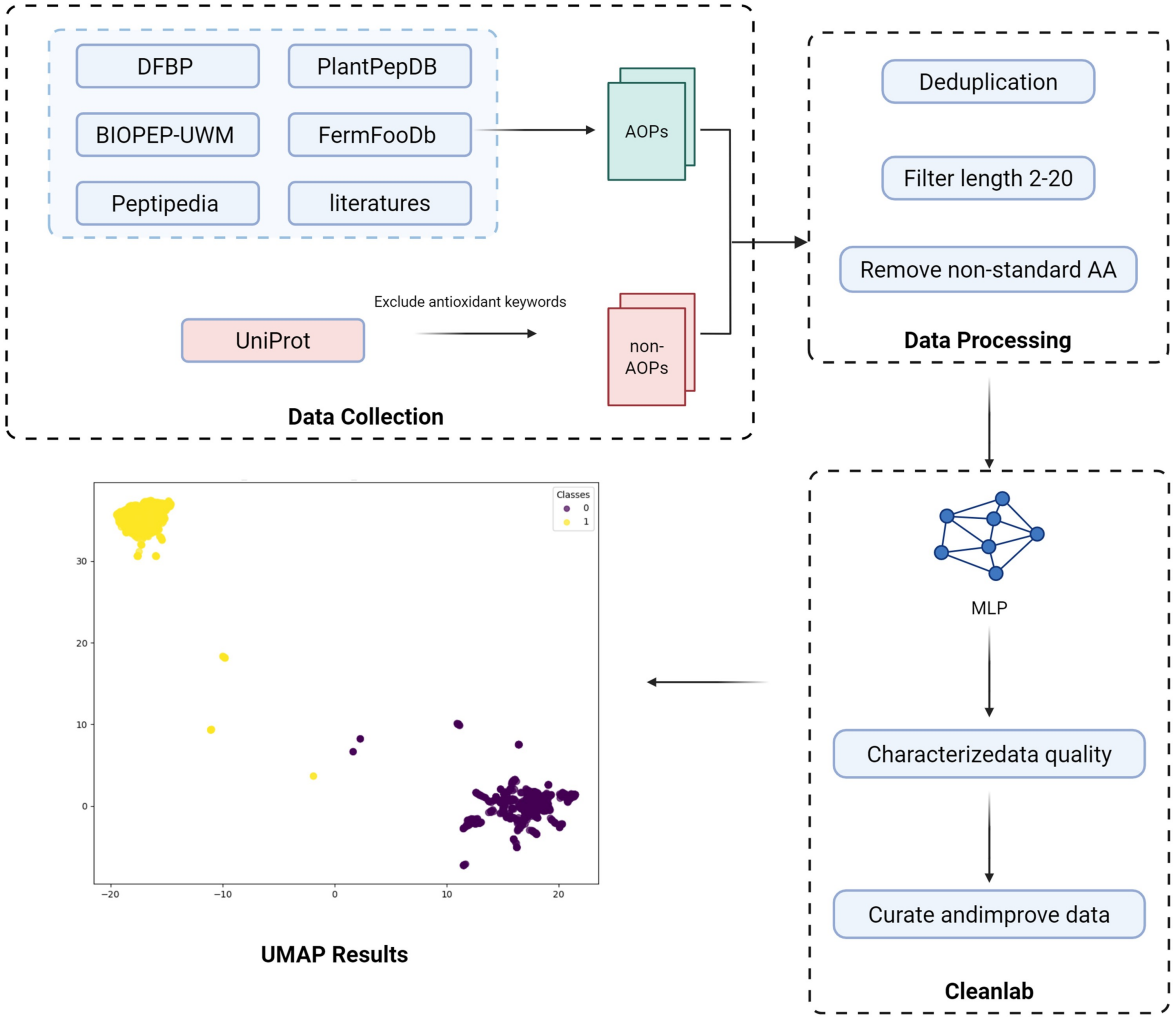
Workflow for high-purity antioxidant peptide dataset construction and validation. Data Collection: Positive samples (AOPs) were gathered from various specialized databases and literature, while negative samples were sourced from UniProt. Data Processing: The raw sequences underwent several cleaning steps, including deduplication, length filtering (2–20 AAs), and removal of non-standard amino acids. Cleanlab: A confident learning algorithm (CleanLab) was applied to programmatically identify and remove potential label noise, thus curating and enhancing the final data quality. UMAP Results: The final cleaned dataset was visualized using UMAP, confirming a clear and distinct separation between the AOP class (yellow) and non-AOP class (purple), which validates the effectiveness of the data purification process.

The foundation of any robust machine learning model is a high-quality dataset. Our data construction process, outlined in Fig. 2, is a multi-stage effort designed to maximize data purity and reliability. We began by aggregating a comprehensive set of potential antioxidant peptides (AOPs) from multiple public databases (including DFBP (Qin *et al*. 2022), BIOPEP-UWM (Minkiewicz *et al.* 2019), Peptipedia (Quiroz *et al.* 2021), AODB (Deng *et al.* 2023), PlantPepDB (Das *et al*. 2020), FermFooDb (Chaudhary *et al*. 2021)) and literature sources. Negative data were retrieved from the UniProt database via the UniProt REST API for peptides of 2–20 amino acids, excluding entries containing antioxidant-related keywords (antioxidant, antioxidative, anti-oxidant, anti-oxidative, oxidative stress, free radical, free radicals, radical scavenger, oxidant scavenger, reactive oxygen species, ROS clearance, ROS scavenger, ROS detoxification, ROS inhibition, metal chelation, metal chelator, metal-binding, and chelating activity). Sequences containing non-standard residues in both the positive and negative datasets, as well as the negative data that were duplicated with the positive dataset, were removed.

This raw data then underwent rigorous preprocessing, which included deduplication, filtering to retain peptides within the desired 2–20 amino acid length range, and removal of any sequences containing non-standard amino acids. Recognizing that label noise is a common impediment in biological datasets, we employed CleanLab (Northcutt *et al.* 2021), a confident learning algorithm, to programmatically identify and remove likely mislabeled samples. This crucial step programmatically curated and improved the data quality, yielding a final, high-purity dataset for model training.

To visually confirm the efficacy of this cleaning procedure, we projected the feature embeddings of the final peptide dataset into a two-dimensional space using Uniform Manifold Approximation and Projection (UMAP) (Armstrong *et al.* 2021). As shown in the UMAP results in Fig. 2, the cleaned dataset exhibits a markedly improved separation between the positive (AOPs, class 1, yellow) and negative (non-AOPs, class 0, purple) classes. The physicochemical property analysis (Figure S1, available as supplementary data at *Bioinformatics* online in Supplementary Information) and the consistency check between training and independent sets (Figure S2, available as supplementary data at *Bioinformatics* online in Supplementary Information) further validate the dataset quality. The clear formation of distinct, compact clusters provides strong visual evidence that the noise reduction process was successful, resulting in a high-purity dataset ideal for training a high-performance classifier.

### 3.2 Model development and performance analysis

#### 3.2.1 Framework overview

**Figure 3:**
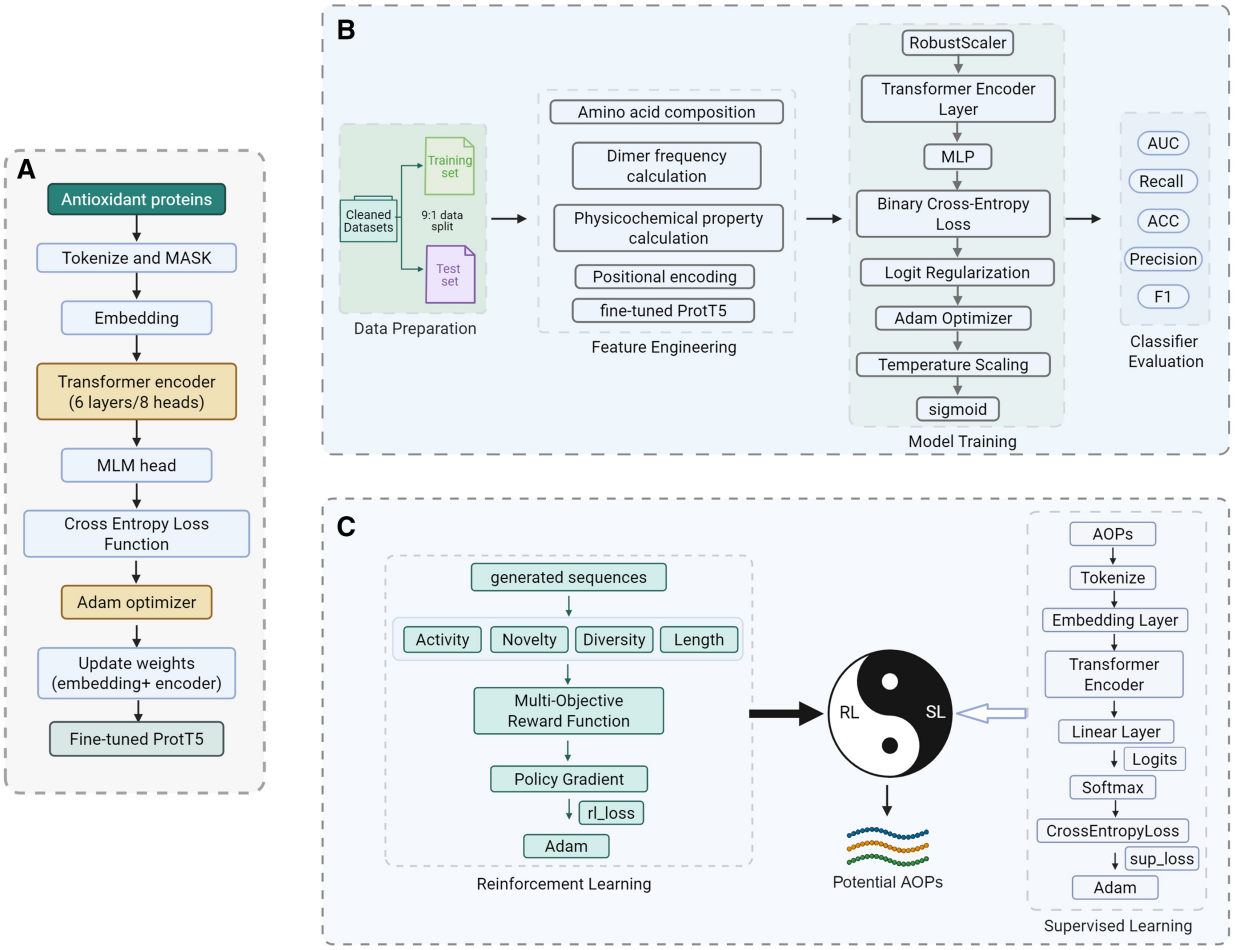
Model Architecture and Technical Implementation. The framework is divided into three core components. (A) Domain-Adaptive Fine-tuning: This flowchart illustrates the process of fine-tuning the ProtT5 model on a dataset of antioxidant proteins via a Masked Language Modeling (MLM) task, creating a specialized foundational model. (B) The Predictor Module (RLP-T5Pred): This diagram shows the workflow for the activity predictor, from data preparation and hybrid feature engineering to the multi-stage model training process for enhanced accuracy and calibration. (C) The Generator Module (RLP-T5Gen): This schematic depicts the “Yin-Yang” training strategy for the peptide generator. It combines SL on known AOPs with an RL loop guided by a multi-objective reward function (activity, novelty, diversity, length) to produce novel, high-potential antioxidant peptides.

Our study introduces the RLAnOxPeptide framework (Fig. 3), designed for state-of-the-art prediction and innovative generation of antioxidant peptides (AOPs). Its architecture features two modules—a predictor (RLP-T5Pred) and a generator (RLP-T5Gen)—both built upon a common foundational language model, ProtT5. ProtT5 is a T5-based large language model pretrained on the extensive UniRef50 database (Suzek *et al.* 2015) of millions of protein sequences. Its extra-large architecture captures complex patterns and long-range correlations, yielding highly informative representations (Yang and Xu 2024). We specialized this model via domain-adaptive fine-tuning on a curated dataset of antioxidant proteins. Using a Masked Language Modeling (MLM) task, the model learned specific sequence characteristics, creating a specialized backbone for both modules.

The first module, the predictor RLP-T5Pred, is a classifier designed to evaluate the antioxidant potential of peptides. Built on the specialized ProtT5 backbone, it undergoes a two-stage training process. It is initially trained with supervised learning (SL) and subsequently refined through a reinforcement learning (RL) co-training stage. This RL phase specifically serves to optimize the classifier’s own decision boundary, using a policy network to improve its ability to discriminate between high and low-activity molecules.

The second module, the generator RLP-T5Gen, is a generative model tasked with creating novel peptide sequences. While it shares the same ProtT5 backbone, its training framework is fundamentally different, incorporating a dynamic balance of two complementary forces akin to the Eastern philosophy of “Tai Chi.” Its strategy combines an initial supervised learning phase (the “Yin” of pattern acquisition) to learn basic AOP patterns, with a subsequent reinforcement learning phase (the “Yang” of goal-oriented innovation). Critically, this RL stage uses the fully-trained RLP-T5Pred as the primary source of its reward signal. It employs the REINFORCE policy gradient algorithm to maximize a multi-objective function that integrates the predictor’s score with objectives for sequence length, diversity, and novelty, thereby guiding the generation of effective new peptides.

#### 3.2.2 Predictive model construction strategy and comparative study

*Model Framework and Regularization Strategy:* To establish a robust predictor serving both as an accurate AOP classifier and a reliable reward function for the downstream generative model, RLP-T5Pred was developed based on the fine-tuned ProtT5 architecture. Critical to its function as a reinforcement learning signal, label smoothing and logit penalty regularization were implemented during supervised training. This strategy mitigates model overconfidence and ensures a well-calibrated probability distribution, thereby providing a stable foundation for generative model training.

*Data Strategy and Internal Validation:* Subsequently, different training set compositions were compared by examining the impact of sequence homology. Five-fold cross-validation indicated that strict redundancy removal (cd-hit 60%) resulted in lower performance (AUC-ROC: 0.9485 ± 0.0149), whereas the strategy of retaining all non-duplicate sequences yielded superior results (AUC-ROC: 0.9650 ± 0.008) as shown in Table S1 and Figure S3, available as supplementary data at *Bioinformatics* online in Supplementary Information. This suggests that retaining diverse sequence patterns—including homologous ones—is more effective for capturing complex AOP features. Meanwhile, the model demonstrated stable convergence, evidenced by a steady decrease in training loss alongside controlled growth of the logit penalty (Fig. S4, available as supplementary data at *Bioinformatics* online). Finally, to interpret the model’s decision-making basis, global feature importance was analyzed using SHAP values (Fig. S5, available as supplementary data at *Bioinformatics* online) and a dipeptide interaction heatmap (Fig. S6, available as supplementary data at *Bioinformatics* online), which highlighted the significant contribution of specific residues such as Tryptophan (W), Lysine (K), and Cysteine (C) to antioxidant activity. These residues, by virtue of their electron-rich indole rings (W), active hydrogen-donating side chains (K), and strongly reducing thiol groups (C), endow peptides with superior electron-donating capacity and radical scavenging potential, which is highly consistent with the recognized structure-activity relationship characteristics of highly active antioxidant peptides (Zou *et al.* 2016).

*Comparison with State-of-the-Art Methods:* Finally, RLP-T5Pred was benchmarked against five existing mainstream prediction tools (AnOxPePred (Olsen *et al.* 2020), AnOxPP (Qin *et al*. 2023), AOPxSVM (Li *et al.* 2025a), PeptideRanker (Mooney *et al.* 2012), and UniDL4BioPep (Du *et al.* 2023)) on an independent test set. RLP-T5Pred significantly outperformed all baseline models as shown in Table 1. Notably, even the RLP-T5Pred (cd-hit60%) model, which performed relatively weakly in our internal validation, achieved an AUC-ROC (0.9239) that far exceeds the best-performing existing tool, UniDL4BioPep (0.7189). Meanwhile, the RLP-T5Pred (all data) model, employing the optimal data strategy (de-duplication only), achieved the most outstanding comprehensive performance, with an AUC-ROC reaching 0.9692 and an F1-Score of 0.9286. Consequently, to maximize the model’s generalization potential and predictive accuracy, the final RLP-T5Pred adopted the training strategy of utilizing all non-duplicate data, that is RLP-T5Pred (all data) model.

**Table 1:** Performance comparison of various models on independent test sets.

Model	AUC-ROC	F1-Score	Precision	Recall	Accuracy
**AnOxPePred**	0.6482	0.5047	0.7941	0.3699	0.5470
**AnOxPP**	0.4682	0.8148	0.7416	0.9041	0.7196
**AOPxSVM**	0.5856	0.7791	0.6768	0.9178	0.6752
**PeptideRanker**	0.4573	0.4754	0.5918	0.3973	0.4530
**UniDL4BioPep**	0.7189	0.7429	0.7761	0.7123	0.6923
**RLP-T5Pred (cd-hit60%)**	0.9239	0.8700	0.8878	0.8529	0.8624
**RLP-T5Pred (all data)**	0.9692	0.9286	0.9167	0.9408	0.9281

a ‟cd-hitX%” indicates datasets where sequences longer than 5 residues were clustered at X% identity threshold to remove redundancy. The first five models (AnOxPePred to UniDL4BioPep) were evaluated on the cd-hit60% independent test set.

To further evaluate the generalizability of RLP-T5Pred, an additional benchmark comparison was conducted using the evaluation setting of a recently reported multimodal deep learning framework for antioxidant peptide prediction (Duy and Srisongkram 2025). In that benchmark, two established external test sets were used for model comparison: the AOPP test set containing 606 sequences and the AnOxPP test set containing 424 sequences. For each test set, sequences overlapping with the corresponding test set were removed from the training data before model development, resulting in two benchmark-specific training sets containing 1,678 and 1,794 sequences, respectively. Following this setting, RLP-T5Pred was retrained separately on the two refined training sets and evaluated on the matched AOPP and AnOxPP test sets using the same evaluation metrics. As summarized in Table S9, available as supplementary data at *Bioinformatics* online, RLP-T5Pred achieved competitive performance on the AOPP test set, with an Accuracy of 0.87, AUROC of 0.92, and MCC of 0.74. On the AnOxPP test set, RLP-T5Pred achieved an Accuracy of 0.96, AUROC of 0.98, MCC of 0.91, Precision of 0.99, and Specificity of 0.99, matching or exceeding the reported benchmark models on several key metrics. These results indicate that the ProtT5-based representation used by RLP-T5Pred remains robust under an external benchmark setting and provides competitive predictive performance compared with recently reported multimodal neural network models.

#### 3.2.3 Performance of RLP-T5Gen

**Figure 4:**
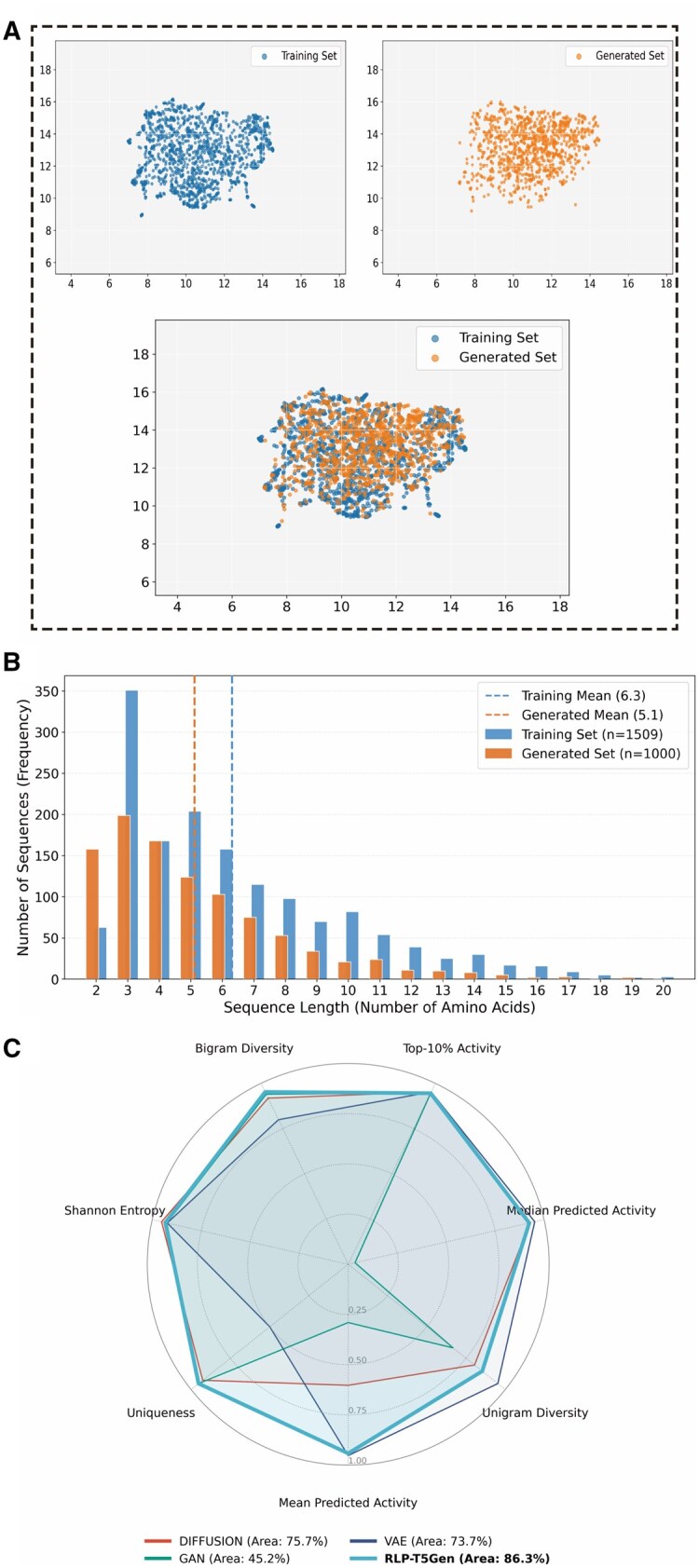
Characterization of novel sequences produced by the RLP-T5Gen generator. Multi-faceted evaluation of 1,000 sequences generated by RLP-T5Gen. (A) UMAP Projection: A visualization showing the generated sequences (orange) in relation to the training set of known AOPs (blue). The generated peptides occupy the same high-activity feature space as known AOPs while also exploring novel regions, indicating both validity and innovation. (B) Length Distribution: A comparison of sequence lengths between the generated sequences and the training set. This demonstrates the model’s ability to adhere to the desired length profile, with mean lengths of 5.1 and 6.3 residues for the generated and training sets, respectively. (C) Comprehensive Model Comparison: A radar chart benchmarking RLP-T5Gen against three state-of-the-art generative baselines: Diffusion, VAE, and GAN. The models are evaluated across seven dimensions, including diversity (Shannon Entropy, Unigram/Bigram), Uniqueness, and Predicted Activity. RLP-T5Gen achieves the highest aggregate performance score (Area: 86.3%), significantly outperforming Diffusion (75.7%), VAE (73.7%), and GAN (45.2%), demonstrating a superior balance between generating diverse, unique, and highly active sequences.

To comprehensively evaluate the performance of the generator RLP-T5Gen, 1000 peptide sequences were generated using this model and compared them with 1000 sequences each generated by three mainstream generative models—Diffusion, Generative Adversarial Network (GAN), and Variational Autoencoder (VAE)—under identical conditions. The results (as shown in Fig. 4 and Table S2, available as supplementary data at *Bioinformatics* online in Supplementary Information) indicate that RLP-T5Gen achieved the optimal balance across multiple key metrics, realizing “controlled creativity.”

First, the UMAP projection (Fig. 4A) provides strong visual confirmation of the generator’s effectiveness. The sequences generated by RLP-T5Gen (orange) not only highly overlap with known AOPs (blue) in the feature space, confirming they possess characteristics of high antioxidant activity, but also occupy blank regions uncovered by the known AOPs (blue), indicating true novelty in exploring new feature spaces. This successful balance is attributed to the dual-stage training strategy: supervised learning ensures biological plausibility, while reinforcement learning drives innovative exploration. The evolution of the training loss (Fig. S7, available as supplementary data at *Bioinformatics* online in Supplementary Information) confirms that the model maintained stability and converged effectively throughout this dynamic optimization process.

Second, the model’s ability to adhere to practical constraints is reflected in the sequence length distribution analysis (Fig. 4B). The length distribution of generated sequences (mean length 5.1) is highly consistent with the training set (mean length 6.3), both showing a trend dominated by short peptides, demonstrating the effectiveness of the multi-objective reward function in length control.

Most importantly, through quantitative comparison with the other three models (Fig. 4C and Table S2, available as supplementary data at *Bioinformatics* online in Supplementary Information), RLP-T5Gen demonstrated superior comprehensive performance. As shown in the radar chart in Fig. 4C, RLP-T5Gen occupies the largest relative polygon area (86.3%), outperforming VAE (73.7%), Diffusion (75.7%), and GAN (45.2%), indicating it is the most balanced across all metrics. Specific numerical analysis shows:

Activity Prediction: RLP-T5Gen’s mean predicted antioxidant activity reached as high as 0.9662, far exceeding GAN (0.2977) and Diffusion (0.6183), and is comparable to VAE (0.9774).

Novelty and Uniqueness: While maintaining high activity, RLP-T5Gen achieved 100% (1.0000) sequence uniqueness, completely avoiding simple rote memorization. In contrast, although VAE had high activity, its uniqueness was only 0.5241, indicating severe overfitting and a tendency to replicate training set sequences; while GAN had high uniqueness, the activity of its generated sequences was extremely low.

Diversity: RLP-T5Gen also performed excellently in diversity metrics. Its Average Unigram Diversity was 0.8840 and Average Bigram Diversity was 0.9916, both outperforming most comparison models, proving that the generated peptides possess rich amino acid compositions and complex sequence patterns.

Taken together, these results demonstrate that the superiority of RLP-T5Gen lies not merely in a larger aggregated radar chart area, but in the capacity of reinforcement learning to transform peptide generation from passive distribution imitation into active goal-directed optimization. Traditional generative models learn the statistical patterns of existing AOPs under fixed loss objectives, often leading to inherent performance trade-offs. For instance, VAE yields peptides with high predicted activity but low uniqueness, indicating strong memorization of training sequences; GAN generates highly novel sequences yet suffers a notable drop in antioxidant activity. By contrast, the RL module of RLP-T5Gen optimizes a unified multi-objective reward function that jointly considers predicted bioactivity, sequence length, internal composition diversity, bigram diversity and structural novelty. Such reward-driven optimization allows the model to explore high-activity regions in the peptide sequence space while avoiding over-memorization of known templates. The supervised learning branch preserves legitimate peptide grammatical patterns, while reinforcement learning further drives functional innovation toward antioxidant propensity. Collectively, the RL framework enables simultaneous coordination of bioactivity, novelty, diversity and practical length control, yielding candidate AOPs that are both biologically plausible and computationally optimized for antioxidant potency.

### 3.3 Peptide design and experimental validation

**Figure 5:**
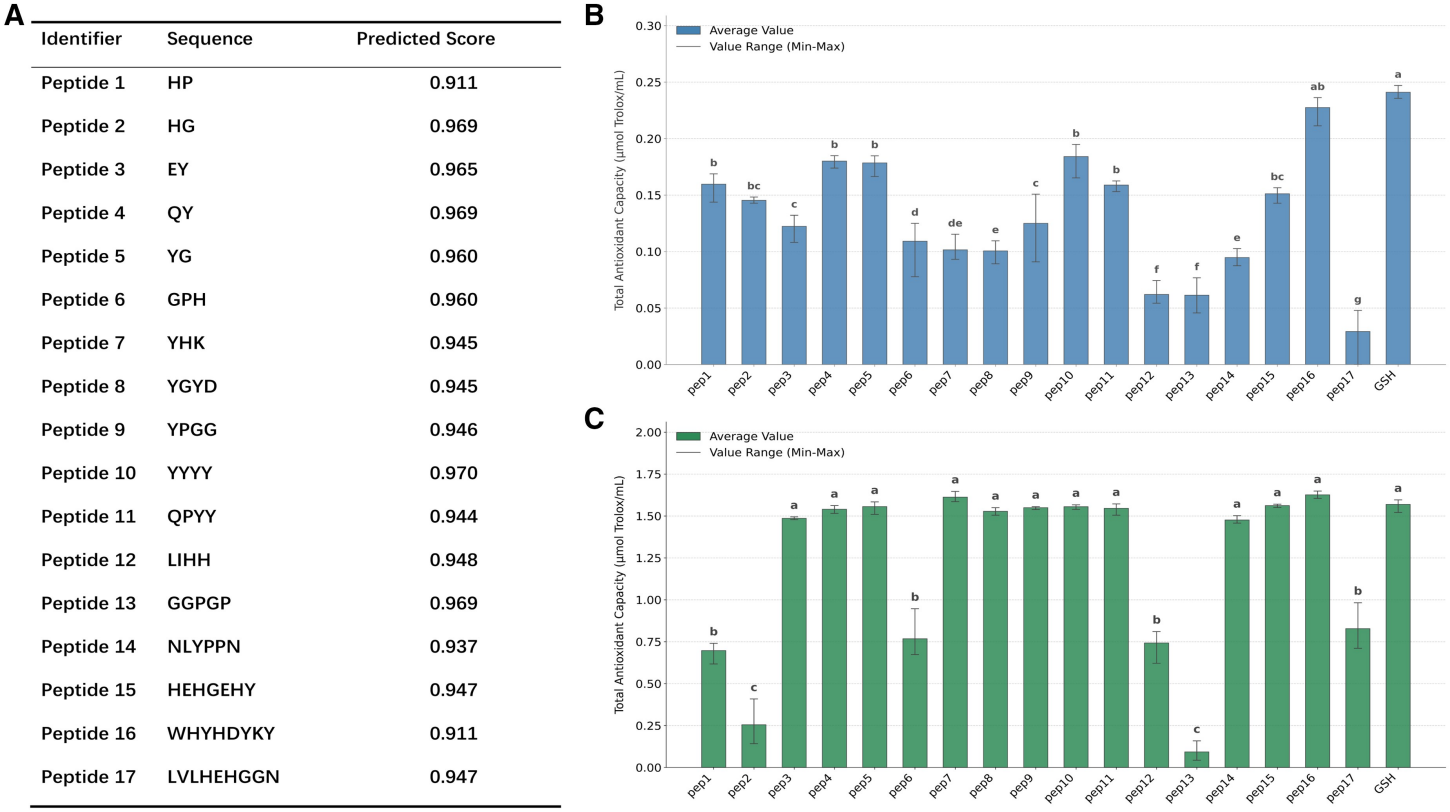
Experimental validation of the antioxidant activity of generated peptides. (A) The 17 designed peptides selected for synthesis, displaying their sequences and predicted activity scores. (B) DPPH radical scavenging activity and (C) ABTS radical cation scavenging activity. Activity is expressed as Trolox equivalents (µmol/L), and data are presented as mean ± *SD* from three independent experiments (*n* = 3). Statistical significance was determined by one-way ANOVA. Different lowercase letters (a–g) above the bars indicate statistically significant differences between groups (*P* < 0.05). Specifically, the lettering follows a ranking order where ‘a’ represents the highest activity level, followed by ‘b’, ‘c’, etc. Conversely, bars sharing a common letter are not significantly different. For example, peptides labeled with ‘a’ (or containing ‘a’ like ‘ab’) exhibited antioxidant potency statistically equivalent to the positive control, Glutathione (GSH).

To close the *in silico-*to*-in vitro loop* and validate our framework’s practical utility, a library of 1,000 novel peptide sequences was generated through the RLAnOxPeptide framework. To align with the training set’s concentration on sequences shorter than 10 amino acids, a diverse set of 17 high-potential candidate peptides was selected from this library. These peptides (sequences and predicted scores detailed in Fig. 5A) represented various lengths and sequence characteristics and were subsequently submitted for chemical synthesis to conduct multi-dimensional experimental validation.

Prior to experimental validation, the physicochemical properties of all 17 designed peptides were systematically analyzed (Table S10, available as supplementary data at *Bioinformatics* online). The generated peptides were short sequences of 2–9 amino acids, with molecular weights ranging from 212.21 to 1211.30 Da. Most peptides showed negative GRAVY values, indicating an overall hydrophilic tendency, which is consistent with their good experimental solubility in PBS. Meanwhile, several experimentally active candidates displayed a high proportion of hydrophobic and/or aromatic residues. For example, Pep4, Pep5, Pep10, Pep11, and Pep16 contained 50.0%–100.0% aromatic residues, mainly contributed by tyrosine. Pep10, composed entirely of tyrosine residues, showed 100.0% hydrophobic and aromatic residue content. These features are consistent with established structure-activity relationships of antioxidant peptides, in which aromatic residues such as tyrosine can contribute to free radical scavenging through hydrogen donation, proton transfer, and electron transfer mechanisms. It should be emphasized that these sequences were not manually designed according to predefined antioxidant residue rules. Instead, they were generated by RLP-T5Gen under the guidance of the learned predictor and the multi-objective reward function. The enrichment of tyrosine-containing, hydrophobic, and aromatic residues among several active candidates suggests that the framework captured antioxidant-relevant biochemical patterns from the training data. This observation is further supported by the SHAP-based interpretation of RLP-T5Pred, which identified specific residues as important contributors to antioxidant prediction. These results indicate that RLAnOxPeptide learned and enriched sequence features associated with antioxidant activity during generation, rather than relying on manual residue selection.

First, to comprehensively assess their fundamental antioxidant capacity, we used two widely accepted chemical assays: the DPPH radical scavenging assay and the ABTS radical cation scavenging assay. The experimental results powerfully confirmed the efficacy of the RLAnOxPeptide framework. The generated peptides showed potent, broad-spectrum efficacy. In the DPPH assay, more than half of the peptides displayed an antioxidant capacity exceeding 50% of the potent positive control, glutathione (GSH), with the top-performing candidate (Pep16) achieving activity statistically equivalent to GSH (Fig. 5B). This exceptional performance was further amplified in the ABTS assay, where 11 of the 17 peptides (Pep3, 4, 5, 7, 8, 9, 10, 11, 14, 15, and 16) demonstrated antioxidant capacity statistically on par with, or even trending higher than, GSH (Fig. 5C).

**Figure 6:**
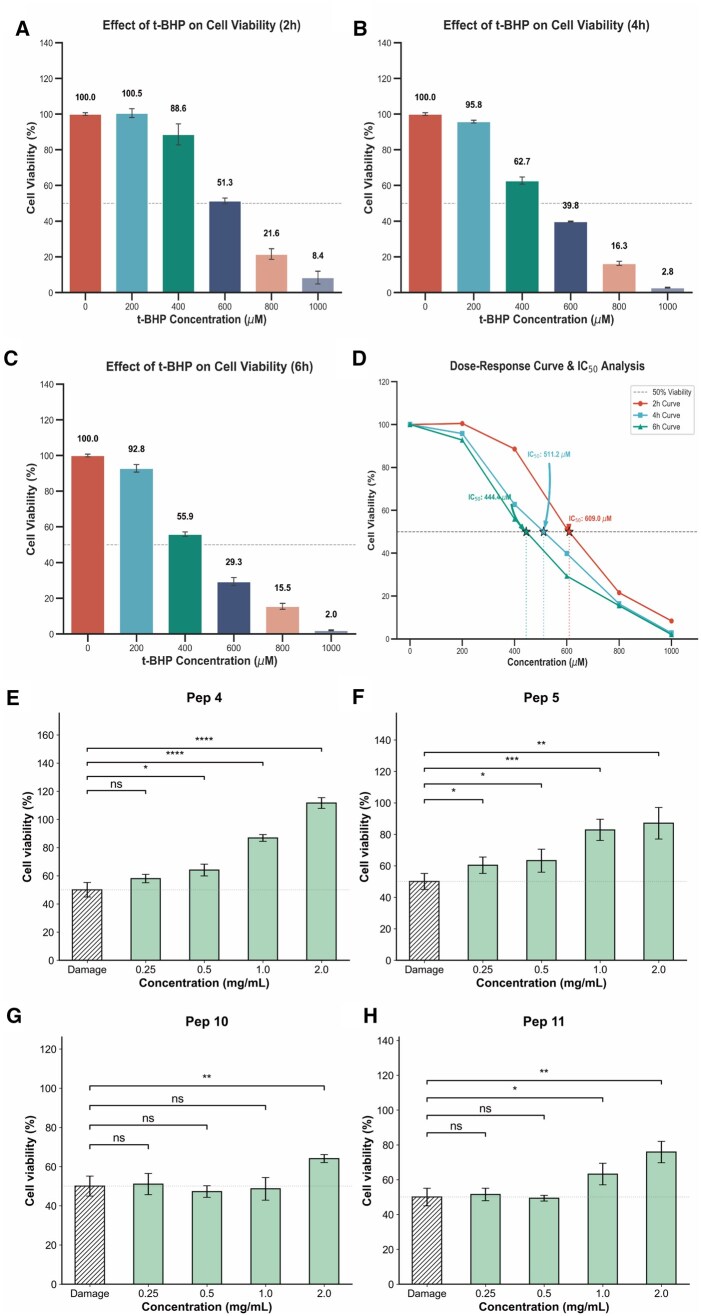
Establishment of the oxidative stress model and evaluation of the cytoprotective effects of candidate peptides in HepG2 cells. (A–D) Optimization of the t-BHP-induced oxidative damage model. Panels (A–C) display the dose-dependent decrease in cell viability after exposure to varying concentrations of t-BHP (0–1000 µM) for 2 hours, 4 hours, and 6 hours, respectively. Panel (D) summarizes these viability curves to determine the half-maximal inhibitory concentration (IC_50_) for each time point (IC_50_ values: 609.0 µM for 2 h, 511.2 µM for 4 h, and 444.4 µM for 6 h), establishing the optimal conditions for subsequent protection assays. (E–H) Cytoprotective efficacy of the top candidate peptides. HepG2 cells were subjected to oxidative damage (using the established IC_50_ condition) and treated with increasing concentrations (0.25, 0.5, 1.0, and 2.0 mg/mL) of Pep4 (E), Pep5 (F), Pep10 (G), and Pep11 (H). The bar charts demonstrate a significant, dose-dependent restoration of cell viability compared to the damage control group (hatched bar). Statistical significance is indicated (*P < 0.05, **P < 0.01, ***P < 0.001, ****P < 0.0001; ns: not significant).

To further evaluate the antioxidant potential of these peptides in biological systems, we initially selected five representative peptides (Pep 4, 5, 10, 11, and 16) that performed most outstandingly in the chemical assays for cell-level validation. However, preliminary experiments revealed that although Pep16 exhibited extremely high free radical scavenging capacity in cell-free systems, it demonstrated significant cytotoxicity towards HepG2 cells, causing cell lysis and death. Consequently, Pep16 was excluded from the subsequent cellular oxidative damage protection experiments, and we ultimately conducted an in-depth evaluation of the remaining four peptides (Pep 4, 5, 10, and 11). In the t-BHP-induced cellular oxidative damage model, we first determined the optimal conditions to establish a model with approximately 50% cell viability loss (Fig. 6A–D and Tables S3 and S4, available as supplementary data at *Bioinformatics* online in Supplementary Information). Subsequently, the protective effect of peptide pretreatment at different concentrations (0.25, 0.5, 1.0, 2.0 mg/mL) on damaged cells was measured using the CCK-8 assay. Table S5, available as supplementary data at *Bioinformatics* online in Supplementary Information details the raw optical density (OD) data for these four peptides across various concentrations.

The results showed that all four peptides significantly alleviated t-BHP-induced oxidative damage in a dose-dependent manner (Fig. 6E–H). Among them, Pep 4 and Pep5 exhibited exceptionally strong cytoprotective activity. As shown in Fig. 6E and F, at a concentration of 2.0 mg/mL, the cell viability of the Pep4 treatment group recovered to over 100% of the control group (significance *P* < 0.0001), and the Pep5 treatment group also recovered to over 80%, indicating that they can effectively maintain cell survival under oxidative stress. Pep10 and Pep11 also showed significant protective effects at high concentrations (Fig. 6G and H).

The consistent success achieved in both chemical assays and cell experiments provides compelling evidence that our framework can robustly identify highly effective and functionally relevant sequences from the vastness of peptide space. This validates that RLAnOxPeptide is capable of designing not only molecules with chemical scavenging abilities but also antioxidant peptides with actual biological protective functions through subsequent screening.

## 4 Conclusion

In this study, we developed RLAnOxPeptide, an integrated computational framework that combines Transformer-based deep learning and reinforcement learning for the efficient prediction and innovative design of antioxidant peptides. The framework consists of two core components: a highly optimized antioxidant peptide predictor (RLP-T5Pred) and an antioxidant peptide sequence generator (RLP-T5Gen).

Results demonstrate that RLP-T5Pred significantly outperforms existing methods in terms of prediction accuracy and robustness, achieving an AUC-ROC of 0.9692 and an F1-Score of 0.9286 on the independent test set. Furthermore, by incorporating strategies such as label smoothing and temperature scaling, we optimized the smoothness of the predicted probability distribution, enabling it to serve as a high-quality reward function for guiding generative tasks. RLP-T5Gen, guided by the optimized classifier and a multi-objective reward function through a “Yin-Yang” iterative training strategy, successfully achieved “controlled creativity.” Compared to mainstream generative models such as GAN, VAE, and Diffusion, RLP-T5Gen achieved the optimal balance between sequence novelty, uniqueness, and diversity while maintaining high predicted activity.

Experimental validation powerfully confirmed the practical utility of the framework. In DPPH and ABTS chemical assays, most designed peptides exhibited activity, with Pep16 demonstrating exceptional free radical scavenging capacity comparable to the benchmark, glutathione (GSH). More importantly, we further validated the biological function of candidate peptides in a t-BHP-induced HepG2 cellular oxidative damage model. Although Pep16 was excluded due to cytotoxicity, other selected peptides (particularly Pep4 and Pep5) showed significant, dose-dependent cytoprotective effects at non-toxic concentrations, effectively enhancing cellular resistance to oxidative damage.

In summary, the RLAnOxPeptide framework represents a significant advancement in AI-driven antioxidant peptide design. Compared to existing approaches, it offers three key advantages: (1) the domain-adapted ProtT5 backbone captures complex long-range sequence dependencies and biochemical context that fixed-length hand-crafted feature descriptors cannot represent, enabling superior generalization; (2) the calibrated predictor–trained with label smoothing and logit penalty regularization–provides a reliable, well-calibrated reward signal for the RL-based generator, preventing the instability that arises when overconfident classifiers are used as reward functions; and (3) the Yin-Yang iterative co-training strategy overcomes the key failure modes of VAE (distributional collapse) and GAN (mode collapse) by balancing chemical validity with directed sequence innovation. Nevertheless, we acknowledge several limitations that should be noted. The antioxidant activity predictor is a binary classifier rather than a quantitative regression model, limiting fine-grained potency ranking. The antioxidant potency reported in the chemical assays (DPPH and ABTS) was evaluated at a single concentration point and expressed as Trolox Equivalents, rather than as IC_50_ values derived from complete dose-response curves; while single-concentration screening is standard practice for initial library evaluation in food science and bioactive peptide research, it does not permit precise pharmacological potency ranking. The framework has not yet been extended to structurally complex peptides such as cyclized or post-translationally modified sequences that may offer enhanced metabolic stability, and all cellular validations were conducted in a single HepG2 cell line model with in vivo efficacy yet to be demonstrated. Future work will focus on: (i) developing a regression-based quantitative predictor for continuous potency ranking; (ii) conducting full dose-response characterization and IC_50_ determination for the top candidates; (iii) extending the framework to modified and cyclic peptides; and (iv) advancing the most promising candidates, particularly Pep4 and Pep5, into in vivo animal models of oxidative stress-related disease to evaluate their therapeutic potential.

## Supplementary Material

btag504_Supplementary_Data

## Data Availability

The datasets generated and/or analysed during the current study, along with model outputs and representative peptide sequences, have been deposited in a public repository. The RLAnOxPeptide framework source code is available at GitHub: https://github.com/changshh/RLAnOxPeptide. An archival snapshot of the code used to perform the experiments described in this manuscript has been deposited in Zenodo with the DOI: 10.5281/zenodo.20078425. An interactive online demonstration is also available via Hugging Face Spaces: https://huggingface.co/spaces/chshan/RLAnOxPeptide.
