# Supplementary material for: RLAnOxPeptide: an integrated framework combining transformer and reinforcement learning for efficient antioxidant peptide prediction and innovative design
Source: Bioinformatics. 2026 Jul 8;42(7):btag504. doi: 10.1093/bioinformatics/btag504 (PMC13401445; doi:10.1093/bioinformatics/btag504)
Supplement: btag504_Supplementary_Data [file btag504_supplementary_data.docx]

# Supplementary Information

RLAnOxPeptide: An Integrated Framework Combining Transformer and reinforcement learning for Efficient Antioxidant Peptide Prediction and Innovative Design

Changsheng Han*^a,b,c^*, Jianda Yue*^a,b,c^*, Yaqi Li*^a,b,c^*, Huanyu Li*^a,b,c^*, Hua Tan*^a,b,c^*, Zhenyu Wang*^a^*, Zhihan Qi*^a^*, Junbao Zhou*^a^*, Zhonghua Liu*^a,b,c,*^*, Ying Wang*^a,b,c,*^*

*^a^The National and Local Joint Engineering Laboratory of Animal Peptide Drug Development, College of Life Sciences, Hunan Normal University, Changsha 410081, Hunan, China*

*^b^Peptide and small molecule drug R&D platform, Furong Laboratory, Hunan Normal University, Changsha 410081, Hunan, China*

*^c^Institute of Interdisciplinary Studies, Hunan Normal University, Changsha 410081, Hunan, China*

*^d^Shanghai Key Laboratory of Green Chemistry and Chemical Processes, School of Chemistry and Molecular Engineering, East China Normal University, Dongchuan Road 500, Shanghai, 200241 China*

^*^Corresponding authors: [wangyin@hunnu.edu.cn](mailto:wangyin@hunnu.edu.cn), [liuzh@hunnu.edu.cn](mailto:liuzh@hunnu.edu.cn)


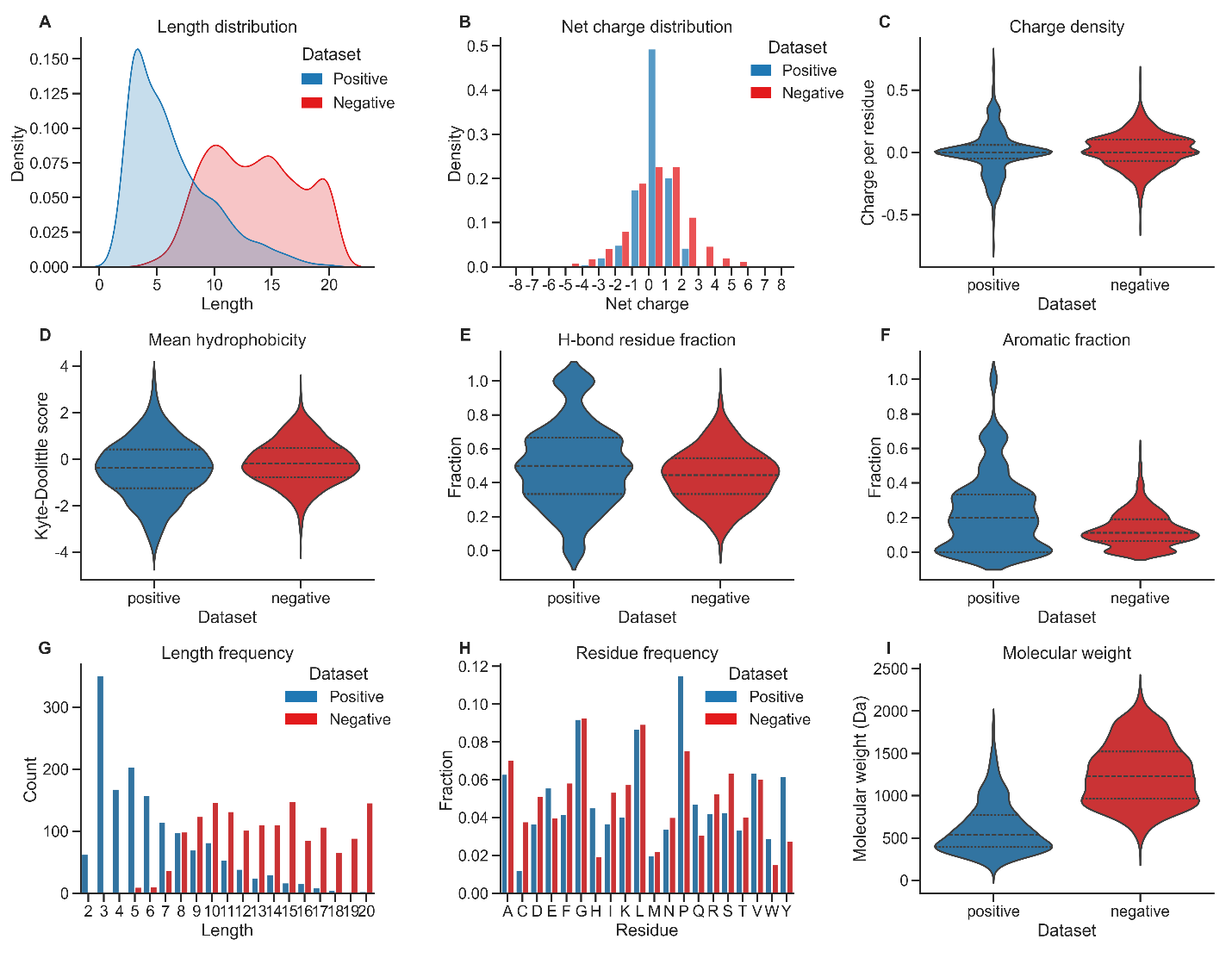


**Figure S1 | Comparison of physicochemical properties between Positive and Negative datasets.** (A) Length distribution. (B) Net charge distribution. (C) Charge density per residue. (D) Mean hydrophobicity (Kyte-Doolittle score). (E) H-bond residue fraction. (F) Aromatic fraction. (G) Length frequency. (H) Residue frequency. (I) Molecular weight distribution.


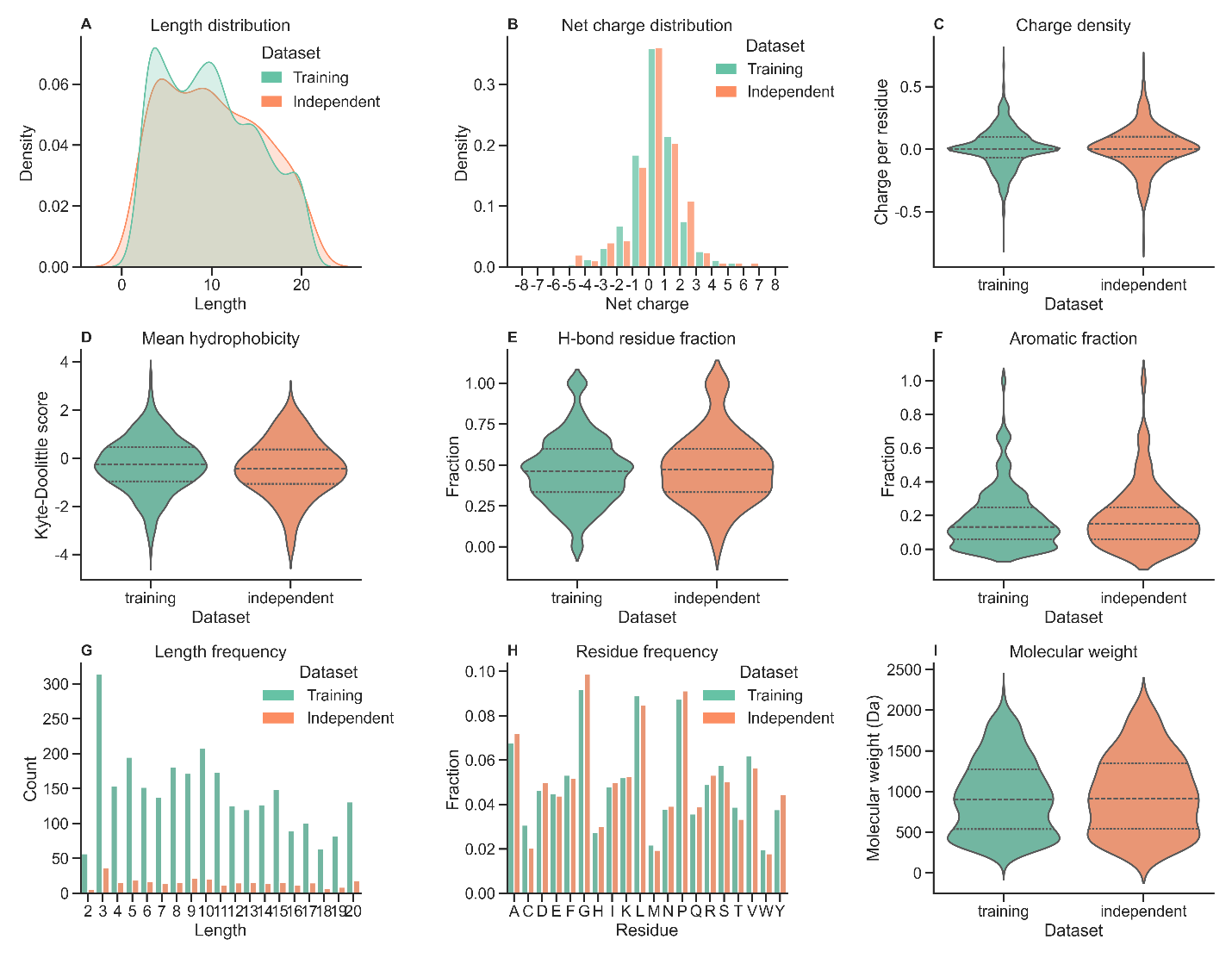


**Figure S2 | Comparison of physicochemical properties between Training and Independent datasets.** (A) Length distribution. (B) Net charge distribution. (C) Charge density per residue. (D) Mean hydrophobicity (Kyte-Doolittle score). (E) H-bond residue fraction. (F) Aromatic fraction. (G) Length frequency. (H) Residue frequency. (I) Molecular weight distribution.


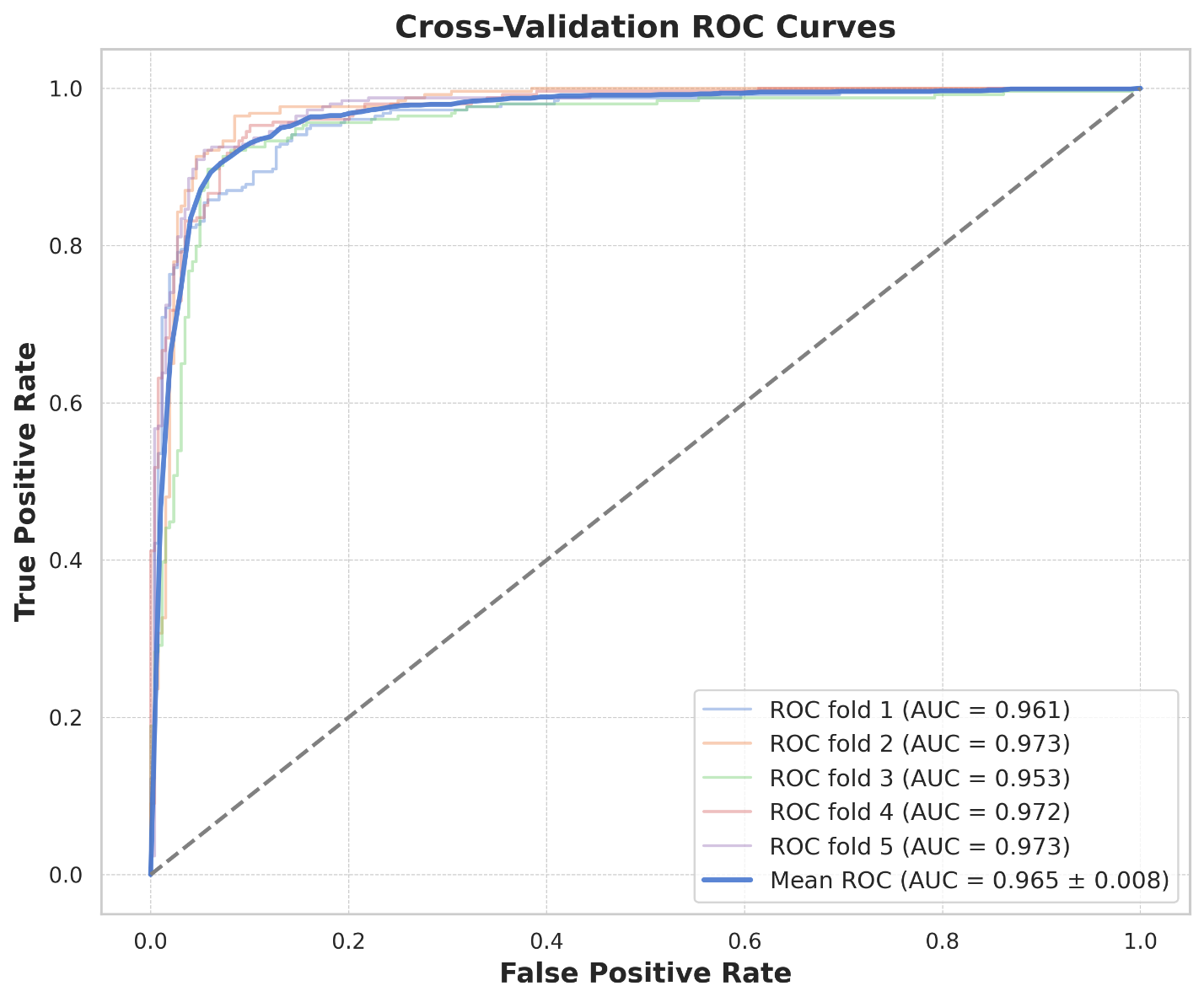


**Figure S3 | Receiver Operating Characteristic (ROC) curves for the 5-fold cross-validation of the RLP-T5Pred model.** The plot displays the individual performance of five cross-validation folds (lighter lines) and the mean ROC curve (thick blue line). The Area Under the Curve (AUC) for each fold ranges from 0.953 to 0.973. The model achieved a mean AUC of 0.965 with a standard deviation of 0.008. The high overlap of the curves and the low standard deviation indicate the robust performance and stability of the RLP-T5Pred predictor across different data partitions.


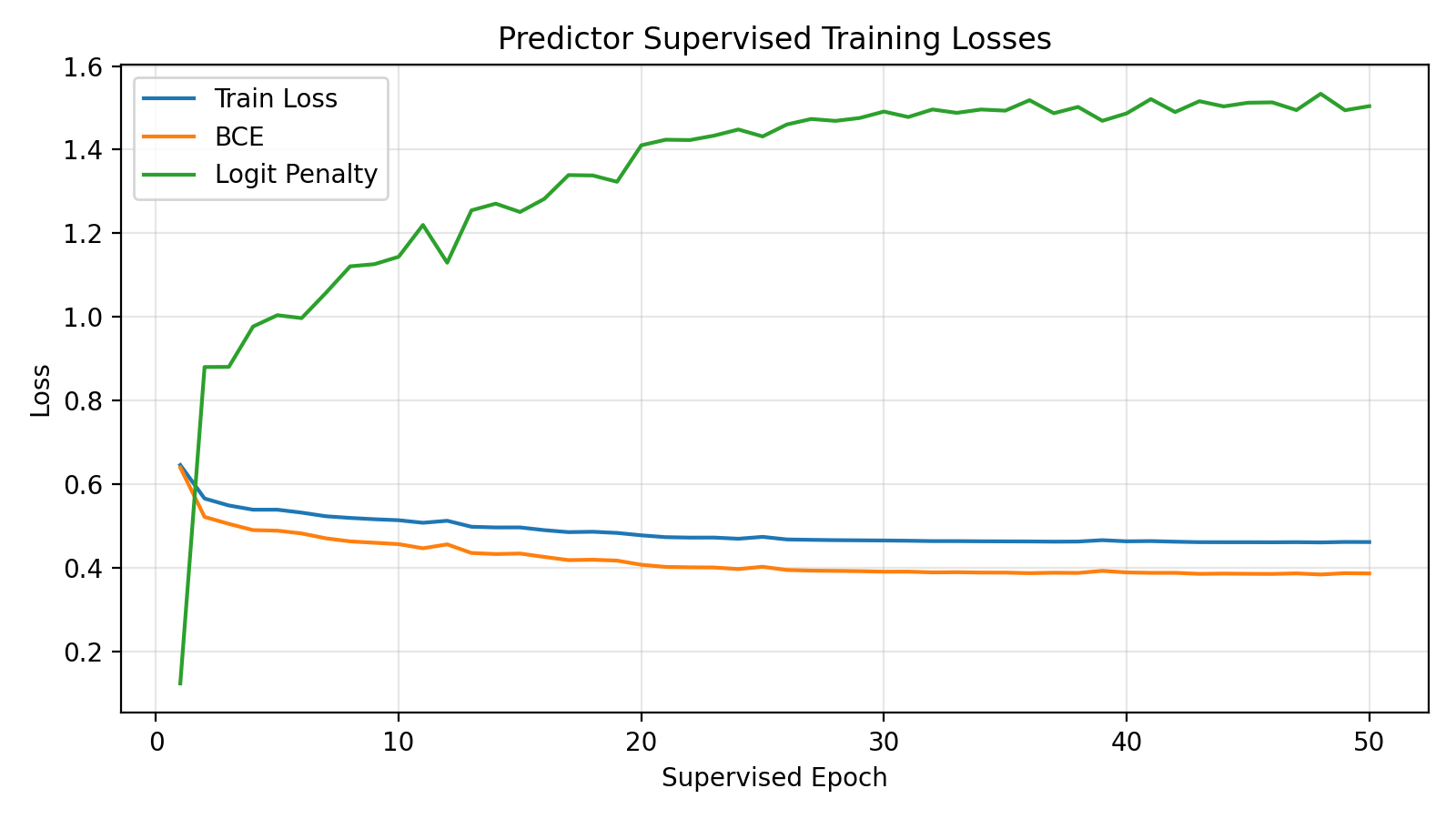


**Figure S4 | Supervised training loss curves for the antioxidant predictor.** The plot illustrates the Total Train Loss (blue), Binary Cross-Entropy (BCE) Loss (orange), and Logit Penalty (green) during the supervised learning phase. The Train Loss decreases steadily, while the Logit Penalty increases to prevent overfitting as model confidence grows.


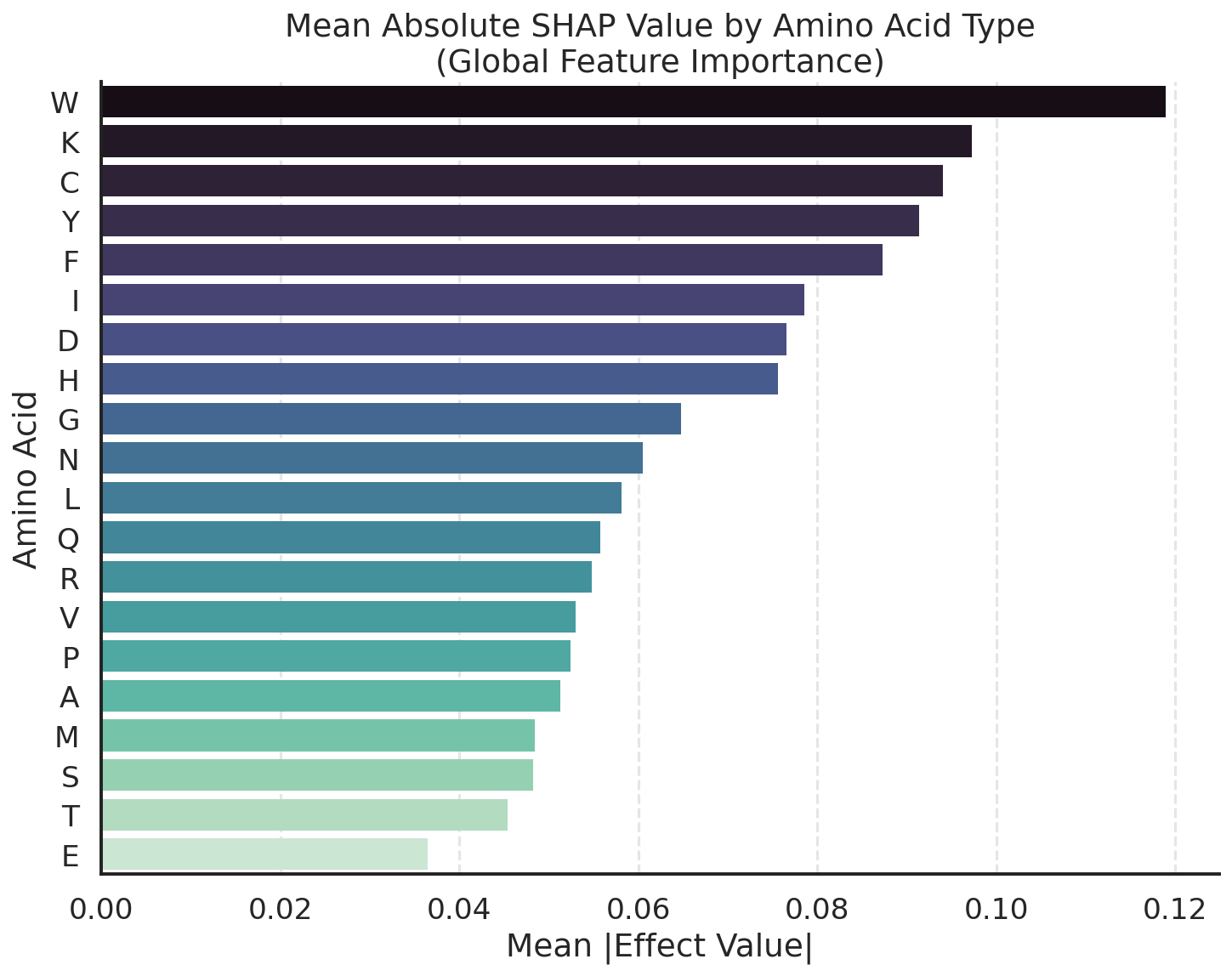


**Figure S5 | Global feature importance based on Mean Absolute SHAP Values.** The bar chart ranks amino acid types by their contribution to the model's predictions, with Tryptophan (W), Lysine (K), and Cysteine (C) showing the highest impact.

**
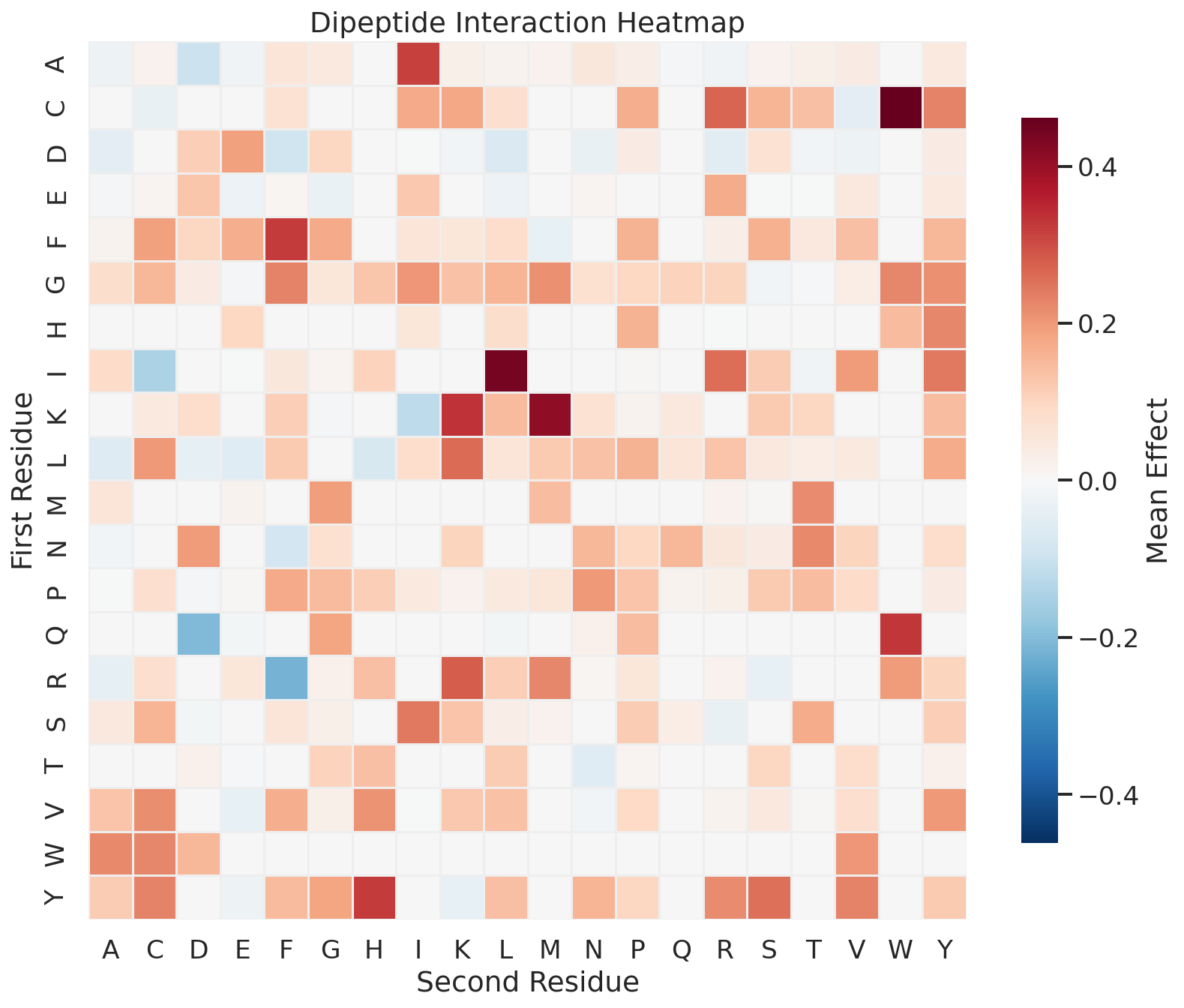
**

**Figure S6 | Dipeptide Interaction Heatmap.** The heatmap visualizes the mean effect of dipeptide combinations (First Residue vs. Second Residue) on the model's output, with red indicating a positive effect and blue indicating a negative effect.


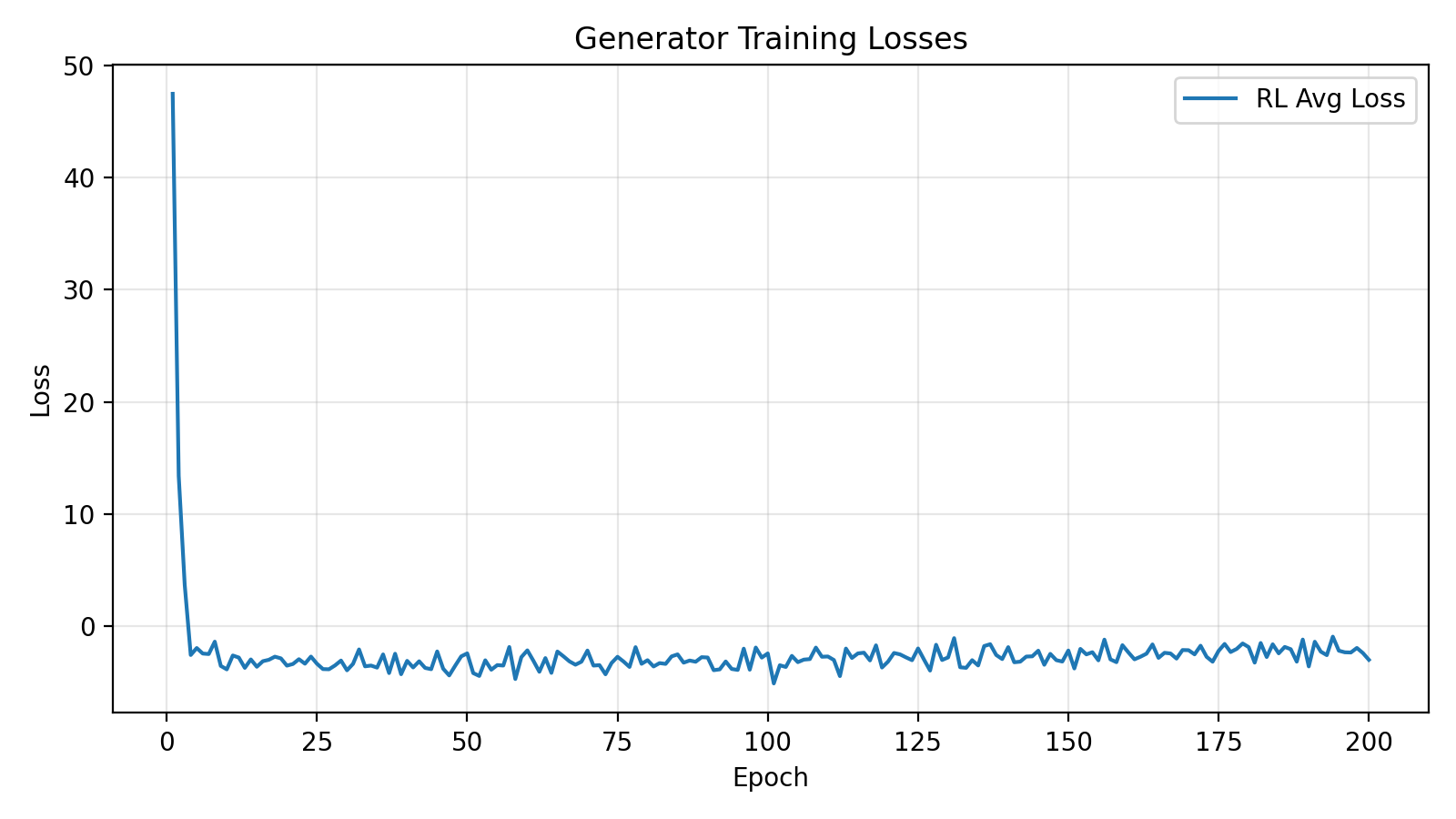


**Figure S7 | Evolution of Reinforcement Learning (RL) loss for the peptide generator over 200 epochs.** The blue line represents the average RL loss. The negative values arise from the optimization objective formulation, where maximizing reward and policy entropy is transposed into minimizing their negative values. Specifically, the persistent negative loss is driven by the entropy regularization term, indicating that the model successfully maximizes sequence diversity (entropy) alongside generation rewards, effectively preventing mode collapse throughout the training process.

**Figure S8 | Mass spectrometry report of Pep1.**

**Figure S9 | Mass spectrometry report of Pep2.**

**Figure S10 | Mass spectrometry report of Pep3.**

**Figure S11 | Mass spectrometry report of Pep4.**

**Figure S12 | Mass spectrometry report of Pep5.**

**Figure S13 | Mass spectrometry report of Pep6.**

**Figure S14 | Mass spectrometry report of Pep7.**

**Figure S15 | Mass spectrometry report of Pep8.**

**Figure S16 | Mass spectrometry report of Pep9.**

**Figure S17 | Mass spectrometry report of Pep10.**

**Figure S18 | Mass spectrometry report of Pep11.**

**Figure S19 | Mass spectrometry report of Pep12.**

**Figure S20 | Mass spectrometry report of Pep13.**

**Figure S21 | Mass spectrometry report of Pep14.**

**Figure S22 | Mass spectrometry report of Pep15.**

**Figure S23 | Mass spectrometry report of Pep16.**

**Figure S24 | Mass spectrometry report of Pep17.**

**Table S1 | Performance comparison of various models on validation sets.**

| **Model** | **AUC-ROC** | **F1-Score** | **Precision** | **Recall** | **Accuracy** |
| --- | --- | --- | --- | --- | --- |
| RLP-T5Pred  cd-hit60% | 0.9485 ± 0.0149 | 0.9021 ± 0.0196 | 0.9119 ± 0.0224 | 0.8927 ± 0.0215 | 0.8958 ± 0.0210 |
| RLP-T5Pred  cd-hit70% | 0.9468 ± 0.0141 | 0.9036 ± 0.0215 | 0.8933 ± 0.0243 | 0.9152 ± 0.0363 | 0.9011 ± 0.0216 |
| RLP-T5Pred  cd-hit80% | 0.9532 ± 0.0113 | 0.8886 ± 0.0164 | 0.8930 ± 0.0282 | 0.8856 ± 0.0326 | 0.8922 ± 0.0158 |
| RLP-T5Pred  cd-hit90% | 0.9569 ± 0.0113 | 0.8919 ± 0.0098 | 0.8850 ± 0.0082 | 0.8992 ± 0.0180 | 0.8998 ± 0.0084 |
| RLP-T5Pred  cd-hit100% | 0.9570 ± 0.0095 | 0.8917 ± 0.0214 | 0.8919 ± 0.0330 | 0.8925 ± 0.0263 | 0.9028 ± 0.0199 |
| RLP-T5Pred  (all data） | 0.9650 ± 0.0080 | 0.9176 ± 0.0160 | 0.9277 ± 0.0318 | 0.9086 ± 0.0174 | 0.9187 ± 0.0173 |

**Table S2 | Comparison of Generated Sequence Parameters across Different Models.**

| **Category** | **Metric** | **RLP-T5Gen** | **DIFFUSION** | **GAN** | **VAE** |
| --- | --- | --- | --- | --- | --- |
| Radar Metric | Mean Predicted Activity | 0.9662 | 0.6183 | 0.2977 | 0.9774 |
|  | Median Predicted Activity | 0.9706 | 0.9730 | 0.0367 | 1.0000 |
|  | Top-10% Activity | 0.9925 | 1.0000 | 0.9937 | 1.0000 |
|  | Uniqueness | 1.0000 | 0.9723 | 1.0000 | 0.5241 |
|  | Shannon Entropy | 4.1826 | 4.2677 | 4.1476 | 4.1388 |
|  | Unigram Diversity | 0.8840 | 0.8331 | 0.6914 | 0.9866 |
|  | Bigram Diversity | 0.9916 | 0.9559 | 0.9786 | 0.8321 |
|  | Relative Polygon Area | 0.8630 | 0.7573 | 0.4524 | 0.7367 |
| Distribution Stat | 25% Quartile (Q1) | 0.9552 | 0.0155 | 0.0009 | 1.0000 |
|  | 75% Quartile (Q3) | 0.9827 | 1.0000 | 0.6957 | 1.0000 |
|  | Std Deviation | 0.0211 | 0.4501 | 0.3913 | 0.1345 |
|  | Min | 0.9001 | 0.0000 | 0.0000 | 0.0000 |
|  | Max | 0.9990 | 1.0000 | 1.0000 | 1.0000 |

**Table S3 | Optical Density (OD) measurements for IC_50_ determination assays.** Data shows raw and mean OD values at different peptide concentrations and time points.

| **Time** | **Concentration (µM)** | **Raw OD Values** | **Mean ± SD** |
| --- | --- | --- | --- |
| Control | 0 | 1.66, 1.69, 1.70 | 1.68 ± 0.02 |
| 2 h | 1000 | 0.39, 0.22, 0.23 | 0.28 ± 0.10 |
|  | 800 | 0.57, 0.46, 0.41 | 0.48 ± 0.08 |
|  | 600 | 0.98, 0.94, 0.90 | 0.94 ± 0.04 |
|  | 400 | 1.69, 1.45, 1.39 | 1.51 ± 0.16 |
|  | 200 | 1.68, 1.76, 1.63 | 1.69 ± 0.07 |
| 4 h | 1000 | 0.20, 0.19, 0.18 | 0.19 ± 0.01 |
|  | 800 | 0.39, 0.43, 0.37 | 0.40 ± 0.03 |
|  | 600 | 0.77, 0.76, 0.75 | 0.76 ± 0.01 |
|  | 400 | 1.12, 1.05, 1.16 | 1.11 ± 0.05 |
|  | 200 | 1.60, 1.64, 1.62 | 1.62 ± 0.02 |
| 6 h | 1000 | 0.18, 0.17, 0.19 | 0.18 ± 0.01 |
|  | 800 | 0.34, 0.43, 0.40 | 0.39 ± 0.04 |
|  | 600 | 0.67, 0.59, 0.55 | 0.60 ± 0.06 |
|  | 400 | 1.04, 1.01, 0.97 | 1.01 ± 0.03 |
|  | 200 | 1.52, 1.63, 1.57 | 1.57 ± 0.06 |

**Table S4 | Optical Density (OD) measurements of damaged cells after 24-hour peptide treatment at IC_50_ conditions.**

| **Group** | **Concentration (mg/mL)** | **Raw OD Values** | **Mean ± SD** |
| --- | --- | --- | --- |
| Damage | - | 0.58, 0.55, 0.46, 0.49 | 0.52 ± 0.05 |
| Pep 4 | 0.25 | 0.57, 0.63, 0.61 | 0.60 ± 0.03 |
|  | 0.5 | 0.64, 0.71, 0.63 | 0.66 ± 0.04 |
|  | 1 | 0.92, 0.87, 0.90 | 0.90 ± 0.02 |
|  | 2 | 1.16, 1.12, 1.20 | 1.16 ± 0.04 |
| Pep 5 | 0.25 | 0.60, 0.69, 0.59 | 0.63 ± 0.05 |
|  | 0.5 | 0.58, 0.73, 0.66 | 0.66 ± 0.08 |
|  | 1 | 0.84, 0.94, 0.80 | 0.86 ± 0.07 |
|  | 2 | 0.83, 1.02, 0.85 | 0.90 ± 0.10 |
| Pep 10 | 0.25 | 0.47, 0.58, 0.54 | 0.53 ± 0.06 |
|  | 0.5 | 0.46, 0.52, 0.49 | 0.49 ± 0.03 |
|  | 1 | 0.46, 0.48, 0.57 | 0.50 ± 0.06 |
|  | 2 | 0.64, 0.68, 0.67 | 0.66 ± 0.02 |
| Pep 11 | 0.25 | 0.51, 0.58, 0.51 | 0.53 ± 0.04 |
|  | 0.5 | 0.53, 0.51, 0.49 | 0.51 ± 0.02 |
|  | 1 | 0.65, 0.59, 0.72 | 0.65 ± 0.06 |
|  | 2 | 0.80, 0.72, 0.84 | 0.79 ± 0.06 |

**Table S5 | CCK8 Assay Absorbance Data for Peptides at Various Concentrations.**

| Peptide | Replicate | 2 mg/mL | 1 mg/mL | 0.5 mg/mL | 0.25 mg/mL |
| --- | --- | --- | --- | --- | --- |
| Pep 4 | 1 | 1.1584 | 0.9207 | 0.6443 | 0.5676 |
|  | 2 | 1.1159 | 0.8724 | 0.7131 | 0.6284 |
|  | 3 | 1.1950 | 0.9049 | 0.6321 | 0.6066 |
| Pep 5 | 1 | 0.8345 | 0.8357 | 0.5769 | 0.5985 |
|  | 2 | 1.0210 | 0.9363 | 0.7280 | 0.6869 |
|  | 3 | 0.8500 | 0.8024 | 0.6615 | 0.5898 |
| Pep 10 | 1 | 0.6394 | 0.4592 | 0.4568 | 0.4665 |
|  | 2 | 0.6789 | 0.4804 | 0.5178 | 0.5759 |
|  | 3 | 0.6734 | 0.5720 | 0.4941 | 0.5445 |
| Pep 11 | 1 | 0.7980 | 0.6501 | 0.5269 | 0.5126 |
|  | 2 | 0.7172 | 0.5934 | 0.5146 | 0.5768 |
|  | 3 | 0.8423 | 0.7210 | 0.4928 | 0.5112 |
| Negative Control |  | 0.5755 | 0.4604 | 0.5473 | 0.4892 |

**Table S6 | Hyperparameters for ProtT5 Fine-tuning for Feature Extraction.**

| **Category** | **Hyperparameter** | **Value** | **Description** |
| --- | --- | --- | --- |
| Model Architecture | Embedding Dimension | 512 | The dimensionality of the token embeddings. |
|  | Number of Transformer Layers | 6 | Number of encoder layers in the transformer. |
|  | Number of Attention Heads | 8 | Number of parallel attention heads in each layer. |
|  | Dropout Rate | 0.1 | Dropout probability for regularization. |
| Training & Optimization | Optimizer | Adam | Adaptive Moment Estimation optimizer. |
|  | Learning Rate | 1 x 10⁻⁴ | Initial learning rate for the Adam optimizer. |
|  | Number of Epochs | 5 | Total number of training epochs. |
|  | Batch Size | 16 | Number of sequences processed in each training step. |
|  | Max Sequence Length | 512 | Maximum token length for input sequences. |
|  | Masking Probability | 0.15 | Probability of a token being masked for MLM. |
|  | Dataloader Workers | 0 | Number of subprocesses for data loading. |

**Table S7 | Hyperparameters for Antioxidant Predictor Training.**

| **Category** | **Hyperparameter** | **Supervised Phase** | **RL-Enhanced Phase** | **Description** |
| --- | --- | --- | --- | --- |
| Model Architecture | Transformer Layers | 3 | 3 | Number of layers in the internal transformer encoder. |
|  | Transformer Attention Heads | 4 | 4 | Number of attention heads in the internal transformer. |
|  | Transformer Dropout | 0.1 | 0.1 | Dropout rate within the internal transformer. |
| Supervised Optimization | Optimizer | Adam | Adam | The optimizer used for the predictor model. |
|  | Learning Rate | 1 x 10⁻⁴ | 5 x 10⁻⁵ | Initial learning rate for the Adam optimizer. |
|  | Weight Decay | 1 x 10⁻⁵ | 1 x 10⁻⁵ | L2 regularization penalty. |
|  | Batch Size | 64 | 64 | Number of samples per training batch. |
|  | Number of Epochs | 10 (with early stopping) | 30 (with early stopping) | Maximum number of training epochs. |
|  | Early Stopping Patience | 15 | 10 | Epochs to wait for F1-score improvement before stopping. |
|  | Label Smoothing | 0.1 | Not explicitly used | Epsilon for label smoothing regularization. |
|  | Logit Penalty Weight (L2) | 0.05 | 0.1 | Weight for penalizing large logit values. |
| Calibration | Temperature Scaling | Yes | Yes | Post-training calibration of output probabilities. |
|  | Calibrator Optimizer | Adam | Adam | Optimizer for finding the optimal temperature T. |
|  | Calibrator Learning Rate | 0.01 | 0.01 | Learning rate for temperature optimization. |
|  | Calibrator Iterations | 100 | 100 | Number of optimization steps for calibration. |

**Table S8 | Hyperparameters for Peptide Generator Training.**

| **Category** | **Hyperparameter** | **Value** | **Description** |
| --- | --- | --- | --- |
| Backbone Fine-tuning | Learning Rate | 3 x 10⁻⁵ | Fine-tuning learning rate for the generator's backbone. |
|  | Number of Epochs | 3 | Fine-tuning epochs for the generator's backbone. |
| Generator Architecture | Embedding Dimension | 512 | The dimensionality of the generator's token embeddings. |
|  | Number of Transformer Layers | 6 | Number of encoder layers in the generator. |
|  | Number of Attention Heads | 8 | Number of attention heads in the generator's transformer layers. |
|  | Dropout Rate | 0.1 | Dropout probability for regularization in the generator. |
| RL Training & Optimization | Optimizer | Adam | Optimizer for the generator model. |
|  | Learning Rate | 3 x 10⁻⁵ | Learning rate for the generator during RL training. |
|  | Number of Epochs | 35 | Total number of RL training epochs. |
|  | RL Steps per Epoch | 5 | Number of policy update steps within each epoch. |
|  | RL Batch Size | 32 | Number of sequences generated and evaluated per RL step. |
|  | Entropy Coefficient | 0.025 | Weight for the entropy bonus in the RL loss to encourage exploration. |
|  | Gradient Clipping Norm | 1 | Maximum norm for gradients to prevent exploding gradients. |
| Supervised Fine-tuning | Supervised Batch Size | 32 | Batch size for supervised fine-tuning steps on known positive peptides. |
|  | Supervised Training Frequency | Every 1 epoch | Frequency at which a full supervised epoch is run. |
| Sequence Sampling | Sampling Temperature | 1.3 | Controls the randomness of token selection during generation. |
|  | Minimum Generation Length | 2 | The minimum number of amino acids in a generated peptide. |
|  | Maximum Generation Length | 20 | The maximum number of amino acids in a generated peptide. |
|  | Repetition Penalty | 1.6 | Penalty applied to logits of tokens that have already appeared. |
|  | Top-p (Nucleus) Sampling | 0.9 | The cumulative probability mass for nucleus sampling. |
| Reward Function Weights | Target Length (Gaussian Mean) | 10 | The ideal peptide length encouraged by the Gaussian reward component. |
|  | Length Std. Dev (Gaussian) | 5.5 | Standard deviation for the Gaussian length reward. |
|  | Classifier Reward | 0.4 | Weight for the reward from the AntioxidantPredictor. |
|  | Length Reward | 0.6 | Weight for the Gaussian-based length bonus. |
|  | Character Diversity Reward | 0.35 | Weight for the reward based on the ratio of unique amino acids. |
|  | Bigram Diversity Reward | 0.35 | Weight for reward based on unique adjacent amino acid pairs. |
|  | Internal Novelty Reward | 0.3 | Weight for penalizing internal repeats within a sequence. |
|  | Training Set Novelty | 1.0 (binary) | A factor of 1.0 is applied if the sequence is not in the training set (0 otherwise). |

**Table S9 | Benchmark comparison of RLP-T5Pred using the AOPP and AnOxPP evaluation settings.**

| **Models** | **Encoded sequences** | **Accuracy** | **AUROC** | **MCC** | **Precision** | **Sensitivity** | **Specificity** |
| --- | --- | --- | --- | --- | --- | --- | --- |
| *For the AOPP test set (n = 606)* | | | | | | | |
| Stacking NN with meta-model | One-hot encoding | 0.89 | 0.93 | 0.78 | 0.94 | 0.83 | 0.95 |
| Stacking NN without meta-model | One-hot encoding | 0.89 | 0.93 | 0.79 | 0.94 | 0.84 | 0.94 |
| The AOPP | ADCA | 0.90 | – | – | 0.98 | 0.83 | 0.98 |
| RLP-T5Pred (Ours) | ProtT5 + LoRA | 0.87 | 0.92 | 0.74 | 0.90 | 0.83 | 0.90 |
| *For the AnOxPP test set (n = 424)* | | | | | | | |
| Stacking NN with meta-model | One-hot encoding | 0.95 | 0.99 | 0.91 | 0.97 | 0.94 | 0.97 |
| Stacking NN without meta-model | One-hot encoding | 0.94 | 0.98 | 0.89 | 0.96 | 0.93 | 0.96 |
| The AnOxPP | SVHEHS | 0.94 | 0.98 | 0.89 | 0.96 | 0.93 | 0.96 |
| The AnOxPP | One-hot encoding | 0.93 | 0.97 | 0.87 | 0.95 | 0.91 | 0.96 |
| RLP-T5Pred (Ours) | ProtT5 + LoRA | 0.96 | 0.98 | 0.91 | 0.99 | 0.92 | 0.99 |

**Table S10 | Physicochemical properties of the 17 designed peptides.**

| **Peptide** | **Sequence** | **Length (aa)** | **MW (Da)** | **pI** | **Net charge (pH 7.0)** | **GRAVY** | **Hydrophobic Residues (%)** | **Aromatic Residues (%)** |
| --- | --- | --- | --- | --- | --- | --- | --- | --- |
| Pep1 | HP | 2 | 252.27 | 7.85 | 0.09 | -2.40 | 0.0 | 0.0 |
| Pep2 | HG | 2 | 212.21 | 7.85 | 0.09 | -1.80 | 0.0 | 0.0 |
| Pep3 | EY | 2 | 310.31 | 3.29 | -1.00 | -2.40 | 50.0 | 50.0 |
| Pep4 | QY | 2 | 309.32 | 5.94 | -0.00 | -2.40 | 50.0 | 50.0 |
| Pep5 | YG | 2 | 238.24 | 5.94 | -0.00 | -0.85 | 50.0 | 50.0 |
| Pep6 | GPH | 3 | 309.32 | 7.85 | 0.09 | -1.73 | 0.0 | 0.0 |
| Pep7 | YHK | 3 | 446.51 | 9.75 | 1.09 | -2.80 | 33.3 | 33.3 |
| Pep8 | YGYD | 4 | 516.51 | 3.10 | -1.00 | -1.62 | 50.0 | 50.0 |
| Pep9 | YPGG | 4 | 392.41 | 5.94 | -0.00 | -0.93 | 25.0 | 25.0 |
| Pep10 | YYYY | 4 | 670.72 | 5.80 | -0.01 | -1.30 | 100.0 | 100.0 |
| Pep11 | QPYY | 4 | 569.61 | 5.88 | -0.00 | -1.92 | 50.0 | 50.0 |
| Pep12 | LIHH | 4 | 518.62 | 8.00 | 0.18 | 0.48 | 50.0 | 0.0 |
| Pep13 | GGPGP | 5 | 383.40 | 6.01 | -0.00 | -0.88 | 0.0 | 0.0 |
| Pep14 | NLYPPN | 6 | 716.79 | 5.94 | -0.00 | -1.28 | 33.3 | 16.7 |
| Pep15 | HEHGEHY | 7 | 907.90 | 5.74 | -1.73 | -2.61 | 14.3 | 14.3 |
| Pep16 | WHYHDYKY | 8 | 1211.30 | 7.81 | 0.18 | -2.33 | 50.0 | 50.0 |
| Pep17 | LVLHEHGGN | 9 | 975.07 | 6.01 | -0.82 | -0.27 | 33.3 | 0.0 |

Note: MW, molecular weight; pI, theoretical isoelectric point; GRAVY, grand average of hydropathicity. Net charge was calculated at pH 7.0. Hydrophobic and aromatic residue contents are expressed as percentages of the total
